# DNASE1L3 enhances antitumor immunity and suppresses tumor progression in colon cancer

**DOI:** 10.1172/jci.insight.168161

**Published:** 2023-09-08

**Authors:** Wenling Li, Hideki Nakano, Wei Fan, Yuanyuan Li, Payel Sil, Keiko Nakano, Fei Zhao, Peer W. Karmaus, Sara A. Grimm, Min Shi, Xin Xu, Ryushin Mizuta, Daisuke Kitamura, Yisong Wan, Michael B. Fessler, Donald N. Cook, Igor Shats, Xiaoling Li, Leping Li

**Affiliations:** 1 Biostatistics and Computational Biology Branch,; 2 Signal Transduction Laboratory,; 3 Immunity, Inflammation, and Disease Laboratory,; 4 Integrative Bioinformatics Support Group,; 5 Epigenetics and Stem Cell Biology Laboratory, National Institute of Environmental Health Sciences (NIEHS), Research Triangle Park, North Carolina, USA.; 6 Division of Cancer Cell Biology, Research Institute for Biomedical Sciences, Tokyo University of Science, Chiba, Japan.; 7 Department of Microbiology and Immunology, University of North Carolina at Chapel Hill, North Carolina, USA.

**Keywords:** Oncology, Colorectal cancer, Dendritic cells, Inflammatory bowel disease

## Abstract

DNASE1L3, an enzyme highly expressed in DCs, is functionally important for regulating autoimmune responses to self-DNA and chromatin. Deficiency of DNASE1L3 leads to development of autoimmune diseases in both humans and mice. However, despite the well-established causal relationship between DNASE1L3 and immunity, little is known about the involvement of DNASE1L3 in regulation of antitumor immunity, the foundation of modern antitumor immunotherapy. In this study, we identify DNASE1L3 as a potentially new regulator of antitumor immunity and a tumor suppressor in colon cancer. In humans, *DNASE1L3* is downregulated in tumor-infiltrating DCs, and this downregulation is associated with poor patient prognosis and reduced tumor immune cell infiltration in many cancer types. In mice, *Dnase1l3* deficiency in the tumor microenvironment enhances tumor formation and growth in several colon cancer models. Notably, the increased tumor formation and growth in *Dnase1l3*-deficient mice are associated with impaired antitumor immunity, as evidenced by a substantial reduction of cytotoxic T cells and a unique subset of DCs. Consistently, *Dnase1l3*-deficient DCs directly modulate cytotoxic T cells in vitro. To our knowledge, our study unveils a previously unknown link between DNASE1L3 and antitumor immunity and further suggests that restoration of DNASE1L3 activity may represent a potential therapeutic approach for anticancer therapy.

## Graphical abstract

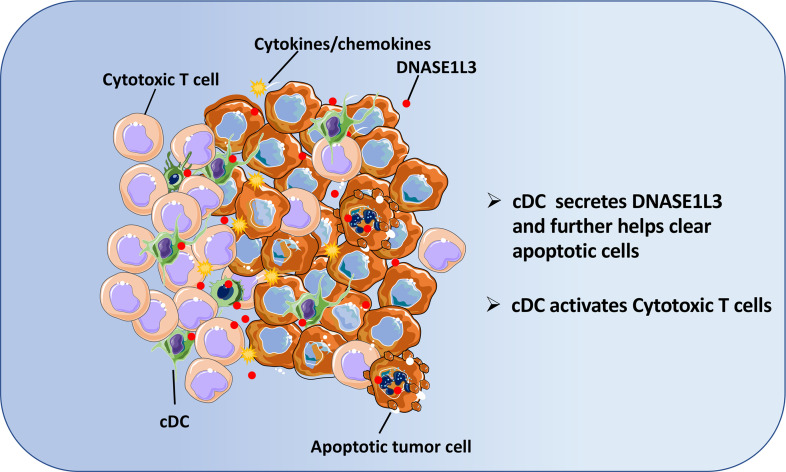

## Introduction

DNASE1L3, a Ca^2+^/Mg^2+^-dependent endonuclease (1), is a secreted protein that, under normal physiological conditions, efficiently digests dietary DNA within the intestinal tract and chromatin released from dead cells (2–4). DNASE1L3 can also digest DNA encapsulated in microparticles released from cells upon activation, malignant transformation, stress, or death (5).

Several lines of evidence suggest that DNASE1L3 is closely associated with systemic immunity. *DNASE1L3* is highly expressed in conventional DCs (cDCs) (6–8) and liver sinusoidal endothelial cells (LSECs) (9), gatekeepers of hepatic immunity (10). It is also moderately expressed in plasmacytoid DCs (pDCs), macrophages, and B cells (8). Deficiency in DNASE1L3 has been associated with development of autoimmune diseases in both humans and mice. Genome-wide association studies have uncovered a link between loss of the functional variant of *DNASE1L3* and autoimmune diseases such as systemic lupus erythematosus (SLE) (6, 11). Immunochip analysis also identified *DNASE1L3* as one of the susceptibility loci for SLE (12). Moreover, autoantibodies against DNASE1L3 are associated with sporadic SLE (13), and patients with a *DNASE1L3* deficiency have elevated DNA levels in plasma (6). Consistently, *Dnase1l3* deficiency in mice leads to rapid development of autoantibodies to double-stranded DNA (dsDNA) and chromatin, along with hyperinflammatory phenotypes including a reduced marginal zone B cell population and increased fractions of monocytes and activated T cells, hyperactivation of germinal centers, early expansion of Tfh cells, elevated plasmablasts in the spleen, and increased incidence of glomerulonephritis (6, 7). Therefore, DNASE1L3 is functionally important for regulating autoimmune responses to self-DNA and chromatin in both humans and mice (6, 7, 14–17).

Despite the evidence of the causal relationship between DNASE1L3, systemic immunity, and autoimmune response, little is known about the involvement of DNASE1L3 in regulation of antitumor immunity. Herein, we systematically investigated the possible role of DNASE1L3 in mediating the interaction between immune cells and cancer cells in both humans and mice using bioinformatics analyses of The Cancer Genome Atlas (TCGA) data sets and various mouse intestinal/colorectal cancer (CRC) models. Our study demonstrates that DNASE1L3 is downregulated in tumors and that this downregulation is associated with poor patient prognosis in many cancer types in humans. Furthermore, *Dnase1l3* deficiency in mice delays tissue recovery after damage, increases chronic inflammation and immune cell dysfunction, impairs antitumor immunity, and enhances tumor progression.

## Results

### DNASE1L3 is downregulated in human tumors, and its downregulation is associated with poor patient survival.

**Figure 1:**
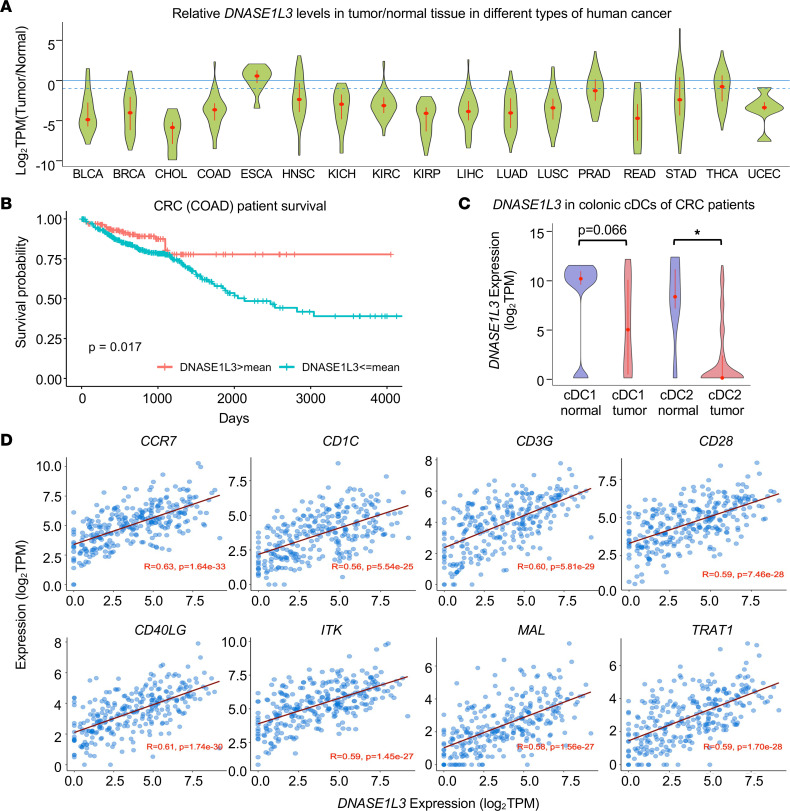
*DNASE1L3* is downregulated in human tumors, and its downregulation is associated with poor patient survival. (**A**) The mRNA levels of *DNASE1L3* are downregulated in multiple types of human cancer. Violin plots of relative *DNASE1L3* expression in paired tumor versus normal tissue for 17 human tumor types based on RNA-Seq data sets from TCGA. BLCA, bladder urothelial carcinoma; BRCA, breast invasive carcinoma; CHOL, cholangiocarcinoma; COAD, cholangiocarcinoma; ESCA, esophageal carcinoma; HNSC, head and neck squamous cell carcinoma; KICH, kidney chromophobe; KIRC, kidney renal clear cell carcinoma; KIRP, kidney renal papillary cell carcinoma; LIHC, liver hepatocellular carcinoma; LUAD, lung adenocarcinoma; LUSC, lung squamous cell carcinoma; PRAD, prostate adenocarcinoma; READ, rectum adenocarcinoma; STAD, stomach adenocarcinoma; THCA, thyroid carcinoma; UCEC, uterine corpus endometrial carcinoma. (**B**) High expression of *DNASE1L3* is associated with increased survival in patients with CRC. Kaplan-Meier survival probability curves for the TCGA colorectal patients (*n* = 597). Survival of the patients with *DNASE1L3* expression level in tumors above the mean are shown in red (favorable), and survival of those with *DNASE1L3* expression level in tumors at or below the mean are shown in blue (unfavorable). *P* value was computed based on the log-rank test of the survival distributions of high and low expression groups. (**C**) *DNASE1L3* is downregulated in cDC1 and cDC2 cells from tumors of patients with CRC compared with those from normal colorectal tissues. scRNA-Seq data sets of human colorectal tumors (18) were analyzed (*n* = 10 for normal cDC1, 36 for tumor cDC1, 36 for normal cDC2, and 64 for tumor cDC2; Mann-Whitney *U* test, *P < 0.05). (**D**) The expression of *DNASE1L3* is positively correlated with that of several immune activation markers, particularly T cell activation markers, in patients with CRC. The mRNA levels of *DNASE1L3* and those of selected immune activation markers from TCGA colorectal tumor RNA-Seq data were analyzed (*n* = 286; Pearson product-moment correlation test).

To evaluate the possible involvement of DNASE1L3 in cancer, we first analyzed RNA-Seq data sets from TCGA and found that the mRNA levels of *DNASE1L3* were significantly downregulated in a wide range of human cancer types compared with their adjacent normal tissues (Figure 1A). Importantly, downregulation of *DNASE1L3* was associated with poor patient survival for many cancer types (Supplemental Figure 1A; supplemental material available online with this article; https://doi.org/10.1172/jci.insight.168161DS1), including CRC (Figure 1B).

*DNASE1L3* is highly expressed in DCs of both humans and mice (6–8) (Supplemental Figure 1B). To test whether the observed downregulation of *DNASE1L3* in human tumors is due to a reduced number of *DNASE1L3*-expressing cells or due to its intrinsic downregulation in those cells, we analyzed a recent public single-cell gene expression profiling study of the CD45^+^ myeloid population in human colorectal tumors (18). This analysis confirmed that *DNASE1L3* has the highest expression in cDC1 and cDC2 among myeloid cells in normal human colonic tissues (Supplemental Figure 1C) and further revealed that its expression in both cDC subtypes, particularly cDC2, was significantly downregulated in human CRC tumors compared with adjacent normal tissues (Figure 1C and Supplemental Figure 1C). These results suggest that the observed downregulation of *DNASE1L3* in human tumors is likely due to its reduced expression in cDCs in tumors.

Further bioinformatic analyses revealed that several immune cell markers, including *CCR7*, *CD40LG*, and *CD3G*, were among the most positively correlated genes with *DNASE1L3* in human colorectal tumors (Figure 1D), indicative of a possible association between *DNASE1L3* expression and antitumor immune activity. Taken together, our in silico analyses of transcriptomic data from human tumors suggest that downregulation of *DNASE1L3* in cDCs may impair antitumor immunity, which in turn contributes to tumor progression.

### Dnase1l3 deficiency in mice results in impaired tissue recovery after DNA damage.

To test the possibility that DNASE1L3 may function as a tumor suppressor, we decided to leverage a previously generated full-body constitutive *Dnase1l3*-deficient (KO) mouse strain (19) in several models of CRC. We confirmed that expression of *Dnase1l3* in all segments of the intestine was dramatically reduced in this *Dnase1l3*-KO mouse strain (Supplemental Figure 2A). *Dnase1l3*-deficient mice were phenotypically normal with normal body weight, and small intestine and colon length were comparable with their WT counterparts (Supplemental Figure 2, B–D). Histological analysis did not reveal any morphological abnormalities in the colon (Supplemental Figure 2E). Expression of cell type–specific marker genes in the colon, such as those for stem cells (*Lgr5*), endocrine cells (*Sst*), and goblet cells (*Muc2*), was also comparable between the 2 genotypes (Supplemental Figure 2F). These data indicate that the GI tracts of young *Dnase1l3*-KO mice show no gross abnormalities at baseline.

**Figure 2:**
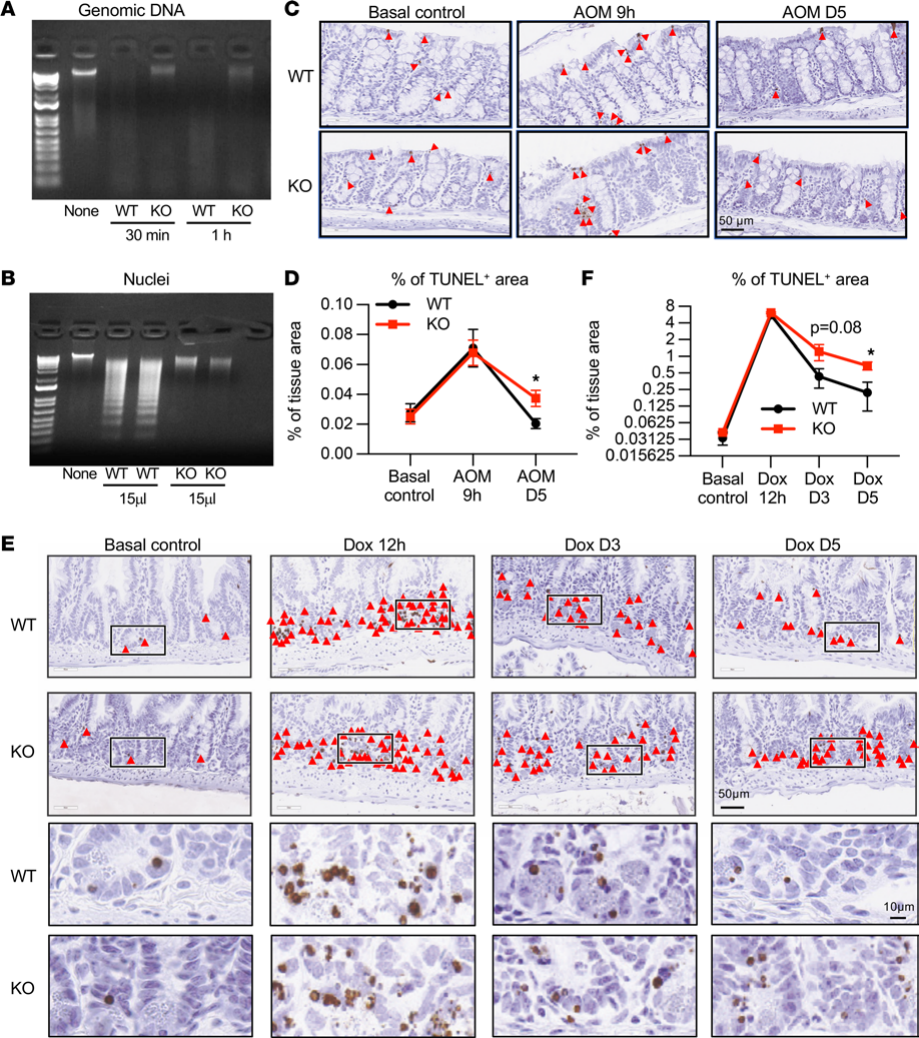
*Dnase1l3*-KO mice display impaired recovery after DNA damage in vitro and in vivo. (**A**) Serum from *Dnase1l3*-KO mice has a reduced ability to digest genomic DNA in vitro. In total, 500 ng genomic DNA was incubated with 5 mL serum isolated from *Dnase1l3* WT and *Dnase1l3-*KO mice. (**B**) Serum from *Dnase1l3*-KO mice has a reduced ability to digest isolated nuclei. In total, 1 × 10^6^ nuclei were incubated with 15 mL serum isolated from *Dnase1l3* WT and *Dnase1l3-*KO mice for 2 hours. (**C** and **D**) *Dnase1l3*-KO mice have a reduced ability to recover in the colon after AOM treatment. Two-month-old female *Dnase1l3* WT and *Dnase1l3-*KO mice were injected with AOM (8 mg/kg), and colonic tissues were isolated 9 hours or 5 days after the injection. Colonic tissue sections were stained by TUNEL. (**C**) Representative images of TUNEL staining of the damaged cell in the colon of *Dnase1l3* WT and *Dnase1l3-*KO mice. Arrow heads, TUNEL^+^ loci. Scale bars: 50 μm. (**D**) Quantification of percentages of TUNEL^+^ signals in **C** (*n* = 4 WT and 4 KO as basal controls; *n* = 5 WT and 6 KO at 9 hours; *n* = 6 WT and 7 KO at D5; Mann-Whitney *U* test, *P < 0.05). (**E** and **F**) *Dnase1l3*-KO mice have a reduced ability to recover in the small intestine after doxorubicin treatment. Two-month-old female *Dnase1l3* WT and *Dnase1l3-*KO mice were injected with doxorubicin (10 mg/kg), and intestine tissues were isolated 12 hours, 3 days, and 5 days after the injection. (**E**) Representative images of TUNEL staining of the damaged cells in the small intestine of *Dnase1l3* WT and *Dnase1l3-*KO mice. Scale bars: 50 μm (top 2 rows) 10 μm (bottom 2 rows). (**F**) Quantification of the percentage of TUNEL^+^ cells in **E** (*n* = 4 WT and 4 KO as basal controls; *n* = 4 WT and 5 KO at 12 hours; *n* = 6 WT and 6 KO at D3; *n* = 5 WT and 6 KO at D5; Mann-Whitney *U* test, *P < 0.05).

Since DNASE1L3 is believed to be a secreted protein (3), we further confirmed the inactivation of this enzyme in KO mice by comparing the ability of serum from *Dnase1l3* WT and *Dnase1l3-*KO mice to digest genomic DNA and chromatin. As expected, serum from *Dnase1l3* WT mice efficiently digested genomic DNA, resulting in DNA smears after 30 minutes of incubation (Figure 2A). In contrast, intact genomic DNA remained visible even after a 1-hour incubation with serum from *Dnase1l3*-KO mice. Similarly, DNA ladders were clearly visible when isolated nuclei were incubated with serum from *Dnase1l3* WT mice, whereas serum from *Dnase1l3*-KO mice failed to generate DNA ladder from nuclei (Figure 2B). Therefore, serum from *Dnase1l3*-KO mice is defective in digesting dsDNA and chromatin DNA in vitro.

*Dnase1l3* deficiency has been shown to hamper the clearance of dsDNA/chromatin in peripheral blood (6, 17). To test the possibility that *Dnase1l3* deficiency may also impair recovery after DNA damage in tissues, we injected *Dnase1l3*-KO and WT mice i.p. with a single dose of azoxymethane (AOM) and evaluated tissue damage in the colon at 2 time points, 9 hours and 5 days (D5), after the injection. TUNEL staining of the colon showed that similar numbers of TUNEL^+^ foci were induced by AOM at the 9-hour early time point in WT and KO mice (Figure 2, C and D). TUNEL^+^ foci were mostly resolved at the D5 after AOM injection in WT mice. However, significantly more TUNEL^+^ foci were present in the colon of the *Dnase1l3*-KO mice at this late time point (Figure 2, C and D). Consistent with this observation, a different DNA double-strand break agent, doxorubicin, induced similar degrees of TUNEL^+^ foci in the small intestine at an early time point (12 hours) in WT and KO mice, but significantly more TUNEL^+^ foci were present in the *Dnase1l3*-KO mice at both D3 and D5 after the injection (Figure 2, E and F). Taken together, these data indicate that *Dnase1l3*-KO mice are impaired in intestinal tissue recovery after DNA damage, which may affect cell homeostasis and subsequent tumorigenesis.

### Dnase1l3 deficiency impairs tissue recovery and increases susceptibility to intestinal cancer formation.

**Figure 3:**
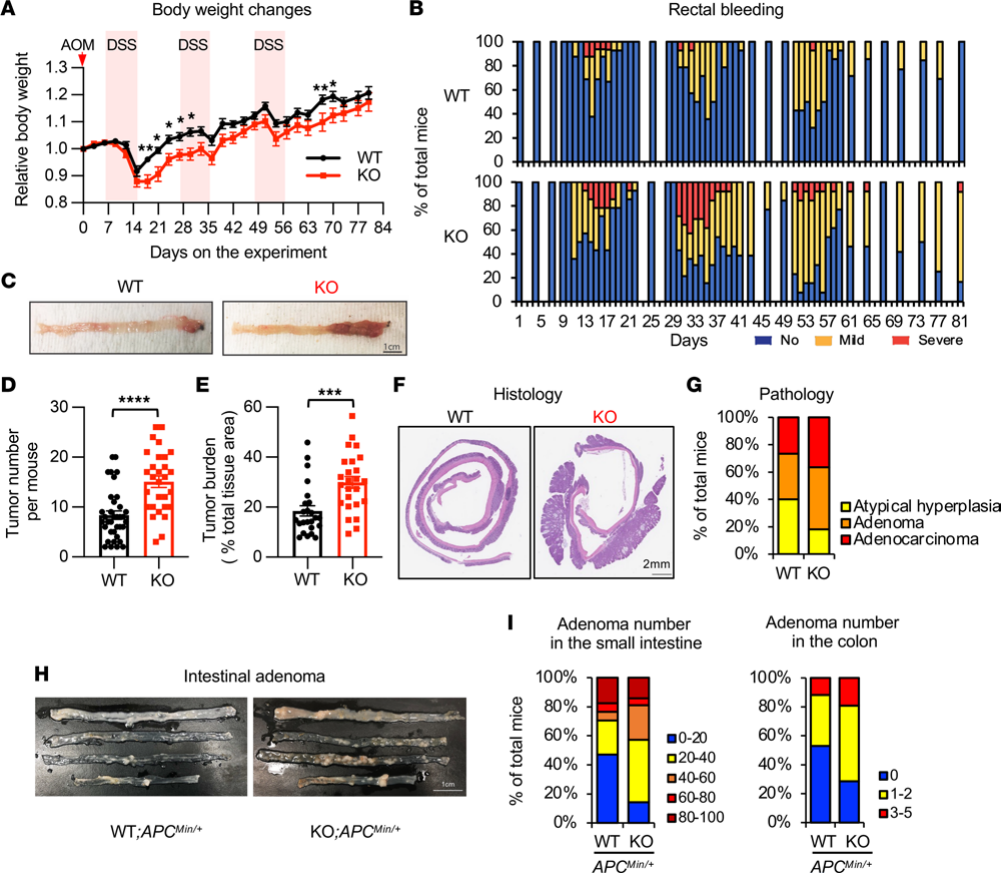
*Dnase1l3* deficiency enhances colon cancer formation in mice. (**A**) *Dnase1l3*-KO mice have increased body weight loss during AOM/DSS treatment. *Dnase1l3* WT and *Dnase1l3-*KO mice were subjected to AOM/DSS CRC procedure with 3 cycles of DSS treatment (peak) as described in Methods (*n* = 15 WT and 15 KO initial mice from 1 experiment; it was independently repeated, and similar result was obtained; Multiple Mann-Whitney *U* tests, *P < 0.05). (**B**) More *Dnase1l3*-KO mice developed severe bleeding and diarrhea than WT mice during the AOM/DSS CRC procedure (*n* = 30 WT and 27 KO from 2 independent experiments). (**C**) Representative images indicating that *Dnase1l3*-KO mice have higher tumor burden than *Dnase1l3* WT mice when treated with 3 cycles of AOM/DSS. Scale bar: 1 cm. (**D**) *Dnase1l3*-KO mice have increased tumor number in the colon after the AOM/DSS CRC procedure (*n* = 36 WT and 29 KO; Mann-Whitney *U* test, ****P < 0.0001). (**E**) *Dnase1l3*-KO mice have increased colon tumor burden. Tumor burden was quantified by Fiji (*n* = 24 WT and 25 KO; Mann-Whitney *U* test, ***P < 0.001). (**F**) Representative H&E colonic images of *Dnase1l3* WT and *Dnase1l3-*KO mice in the AOM/DSS CRC model. Scale bar: 2 mm.(**G**) Colorectal tumors developed in *Dnase1l3*-KO mice are at more advanced stages compared with those in WT mice in the AOM/DSS CRC model. H&E colonic sections from WT and *Dnase1l3*-KO mice (*n* = 15 WT and 13 KO) were evaluated and scored. (**H**) Representative images of intestinal adenoma from WT;APC^min/+^ and KO;APC^min/+^. (**I**) More *Dnase1l3*-KO mice developed adenoma in the small intestine and colon than WT mice in the *APC^min/+^* model (*n* = 15 WT;APC^min/+^ and 21 KO;APC^min/+^).

To directly investigate the possible involvement of DNASE1L3 in suppressing tumorigenesis, we employed a well-established chemically induced CRC model, AOM/dextran sulfate sodium (AOM/DSS) model (20, 21) in *Dnase1l3* WT and *Dnase1l3-*KO mice (Figure 3A, top). In WT mice, the expression of *Dnase1l3* in the colon was markedly induced upon DSS treatment (D10) and remained high during the whole AOM/DSS procedure (Supplemental Figure 3A). Consistent with our observations that *Dnase1l3*-KO mice had significantly more damaged cells in the colon than WT mice after AOM dosing (Figure 2, C and D), *Dnase1l3*-KO mice recovered more slowly than WT mice after the first cycle of DSS treatment, as indicated by greater body weight loss (Figure 3A) and higher incidence of bleeding and diarrhea (Figure 3B and Supplemental Figure 3B). At the recovery phase following the first DSS treatment (D19), *Dnase1l3*-KO mice also had reduced colon length (Supplemental Figure 3C) and impaired recovery of tissue damage (Supplemental Figure 3D), and they showed splenomegaly, as previously reported (6) (Supplemental Figure 3E). Notably, at the final stage of the tumorigenesis process, *Dnase1l3*-KO mice had more tumors, which were also larger than those in WT mice (Figure 3, C–E). Histopathological evaluations of the colons from AOM/DSS-treated mice further revealed that *Dnase1l3*-KO mice had more advanced tumors (adenoma and adenocarcinoma) compared with WT mice, whose tumors had characteristics of atypical hyperplasia (Figure 3, F and G). Therefore, *Dnase1l3* deficiency impairs recovery after short-term treatment and enhanced tumorigenesis after chronic treatment in the AOM/DSS model of inflammation-induced colorectal carcinogenesis. To further confirm these observations, we bred *Dnase1l3*-deficient mice to *APC^min/+^* mice, which develop spontaneous intestinal tumors driven by deregulated β-catenin signaling (22, 23). These animals had more adenomas in the small intestine and colon than littermate *APC^min/+^* mice (Figure 3, H and I), despite having normal gross tissue morphology (Supplemental Figure 4). Collectively, our data from 2 independent colon cancer models demonstrate that *Dnase1l3* deficiency in mice promotes tumorigenesis.

### Dnase1l3 deficiency exacerbates early inflammatory responses but reduces late-stage antitumor immunity.

**Figure 4:**
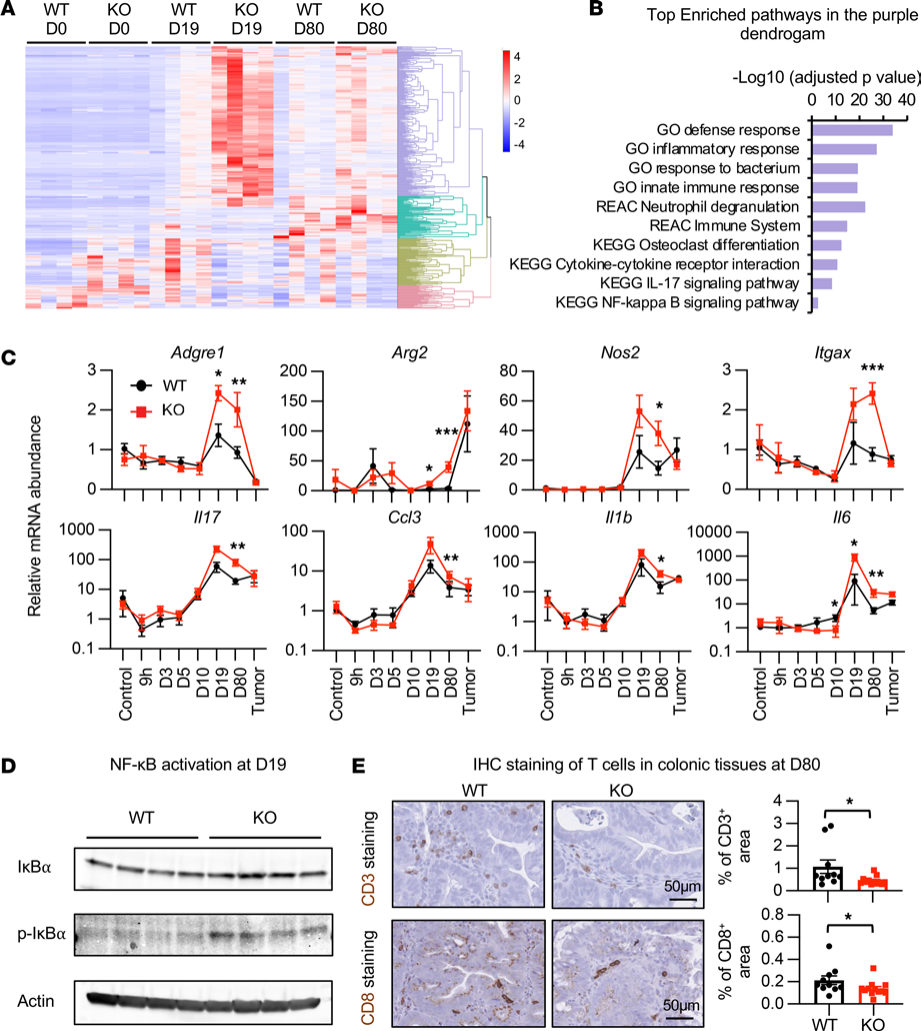
*Dnase1l3* deficiency enhances early-stage inflammation yet reduces late-stage colonic tissue accumulation of T cells in mice. (**A** and **B**) Colonic tissues of *Dnase1l3*-KO mice display increased inflammation during the early stage of AOM/DSS-induced carcinogenesis. Transcriptomes from colonic tissues of WT and *Dnase1l3*-KO mice at D0, D19, and D80 of the AOM/DSS procedure were analyzed by RNA-Seq as described in Methods. (**A**) The dynamics of 4 gene clusters are shown as a heatmap. (**B**) Enriched pathways of the 207 genes shown in the purple cluster. GO, Gene Ontology; REAC, Reactome Pathway Database; KEGG, Kyoto Encyclopedia of Genes and Genome. (**C**) *Dnase1l3*-KO mice have increased expression of various proinflammatory markers at different time points of the AOM/DSS-induced carcinogenesis when analyzed by qPCR (*n* = 4, 5, 5, 6, 10, 6, 12, 5 WT and 4, 6, 7, 6, 6, 12, 10, 2 KO at each time point, respectively; outliers that fall below Q1 − 3.0 × IQR or above Q3 + 3.0 × IQR were removed; Mann-Whitney *U* test, *P < 0.05, **P < 0.01, ***P < 0.001). (**D**) Colonic tissues of *Dnase1l3*-KO mice display increased NF-κB signaling. Western blotting of phospho-IκBα in the colonic tissue samples of the *Dnase1l3* WT and *Dnase1l3-*KO mice at D19 in the AOM/DSS procedure. (**E**) *Dnase1l3*-KO mice have reduced colonic tissue accumulation of T cells at the end stage of the AOM/DSS procedure. Left, representative images for CD3 (upper panels) or CD8 (bottom panels) IHC staining in colorectal tissues from *Dnase1l3* WT and *Dnase1l3-*KO mice after the AOM/DSS CRC procedure. Right, the percentage of CD3^+^ immunostaining areas and CD8^+^ immunostaining areas were quantified from the IHC colonic tissue sections by Fiji (*n* = 10 WT and 12 KO; Mann-Whitney *U* test, *P < 0.05). Scale bar: 50 μm.

To better understand the mechanisms underlying *Dnase1l3* deficiency–induced tumorigenesis, we carried out genome-wide transcriptomic analysis of the whole colon tissue from *Dnase1l3* WT and *Dnase1l3*-KO mice before AOM/DSS treatment (D0), after treatment with 1 cycle of AOM/DSS plus 5-day recovery (D19), and 24 days after treatment with 3 cycles of AOM/DSS (D80) (for the experimental timeline, see Figure 3A). We also performed transcriptomic analysis of the isolated final tumors in parallel. As expected, there were few differentially expressed genes (DEGs; fold change > 2, adjusted *P* [*P*_adj_] < 0.05) between the 2 genotypes at baseline (D0) (Supplemental Table 1). However, during AOM/DSS-induced carcinogenesis, a cluster of 207 genes enriched in pathways of immune and inflammatory responses was dramatically induced in the colon of *Dnase1l3*-KO mice at D19 (Figure 4, A and B, and Supplemental Table 2; the purple cluster, KO). In contrast, the expression of these genes was only modestly induced in the colon of WT mice at the same stage, suggesting that *Dnase1l3* deficiency exacerbates an early-phase inflammatory response in the colon in response to AOM/DSS treatment. Consistent with these observations, expression of the macrophage and DC markers *Adgre1* (F4/80), *Nos2* (Inos), *Arg2* (Arginase-2), and *Itgax* (Cd11c), and various inflammatory cytokines was significantly higher in the colon of *Dnase1l3*-KO mice than those of WT mice, mostly around the first cycle of AOM/DSS treatment (D19) (Figure 4C). Moreover, phosphorylation of IκBα, an indicator of canonical NF-κB activation (24) involved in regulating the expression of several proinflammatory cytokines, was also higher in the colonic tissue of *Dnase1l3*-KO mice compared with that in WT mice (Figure 4D; see complete unedited blots in the supplemental material). Therefore, the colon of *Dnase1l3*-KO mice displayed an early-phase hyperinflammatory response when challenged with AOM/DSS.

Interestingly, expression levels of the proinflammatory genes that were upregulated in the colon of *Dnase1l3*-KO mice at earlier time points did not significantly differ between the final isolated tumors from the 2 genotypes (Figure 4C, tumor). However, RNA-Seq analysis revealed that various genes involved in the type I IFN response (25), including *Ddx60*, *Dhx58*, *Gbp7*, *Gvin1*, *Ifit1bl2*, *Mx2*, *Oas2*, *Oas3*, *Oasl1*, *Oasl2*, and *Slfn4*, were significantly reduced in the final tumors isolated from *Dnase1l3*-KO mice compared with those from WT mice (Supplemental Figure 5), suggestive of a blunted type I IFN response. In addition, *Cd8a*, a marker of cytotoxic T cells, was also found among the 32 downregulated genes (Supplemental Figure 5). Consistently, the numbers of CD3^+^ or CD8^+^ foci were significantly reduced in the colonic tissue from *Dnase1l3*-KO mice at D80 compared with those from WT mice (Figure 4E). Taken together, these observations indicate that the early-phase activation of proinflammatory responses in the colon of *Dnase1l3*-KO mice is followed by a dysfunctional late-phase antitumor immune response characterized by reduced type I IFN signaling and a reduced number of infiltrating cytotoxic T cells (25, 26).

### Dnase1l3 deficiency promotes tumor progression and impairs activity of cDCs in a syngenic tumor model.

**Figure 5:**
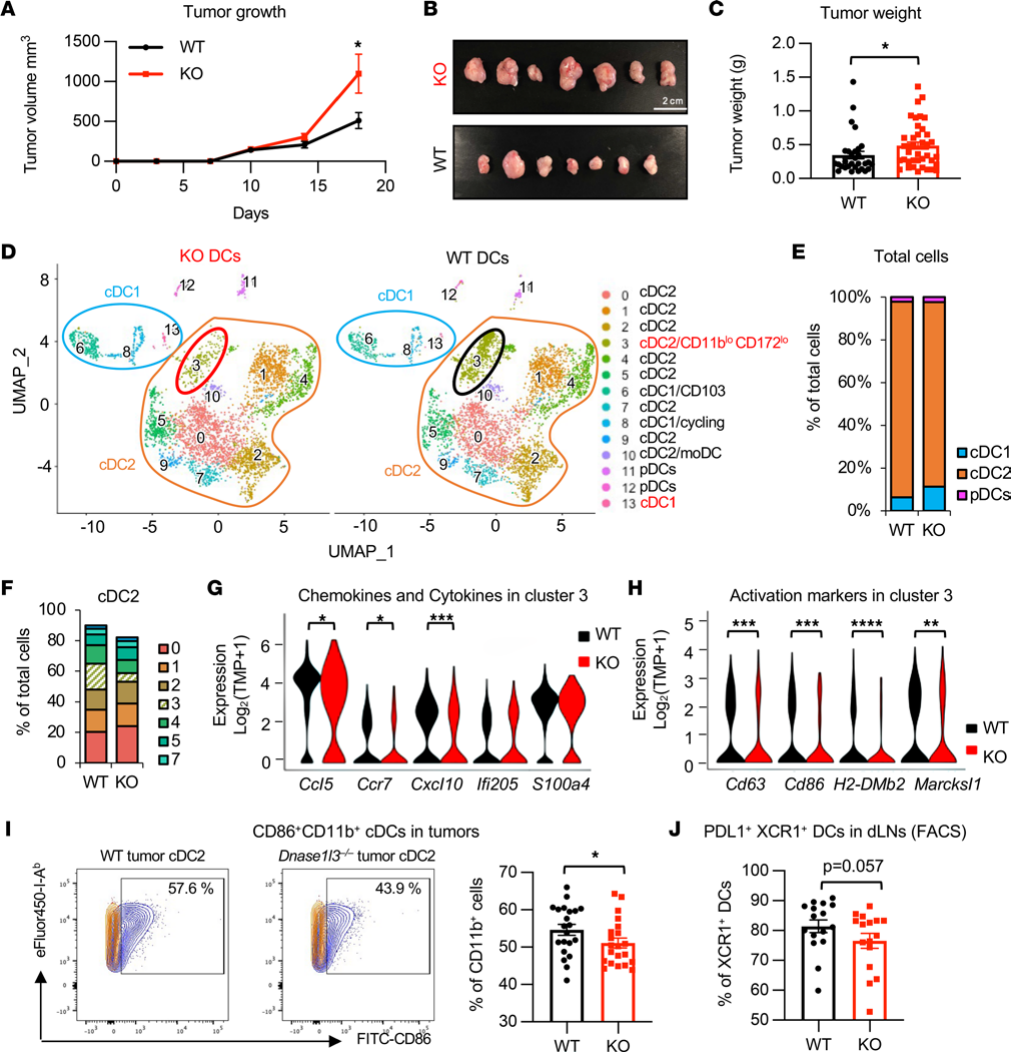
*Dnase1l3* deficiency promotes tumor progression and impairs activity of cDCs in a syngenic tumor model. (**A**) *Dnase1l3* deficiency increases the growth of MC38 tumors. In total, 1 × 10^5^ MC38 colon cancer cells were s.c. injected into 8- to 12-week-old immunocompetent WT or *Dnase1l3*-KO mice (*n* = 10 WT and 11 KO; Mann-Whitney *U* test, *P < 0.05). (**B**) Representative images of final dissected tumors. Scale bar: 2 cm. (**C**) Weights of final MC38 tumors (*n* = 30 tumors from WT and 39 tumors from KO from 3 independent experiments; Mann-Whitney *U* test, *P < 0.05). (**D**) UMAP analysis of cDC single cells sampled from MC38 tumors from *Dnase1l3* WT and *Dnase1l3-*KO mice (analyzed by scRNA-Seq). (**E**) DCs from MC38 tumors in *Dnase1l3*-KO mice have reduced cDC2 but increased cDC1 populations. (**F**) The relative abundance of different subgroups of cDC2 cells. (**G**) *Dnase1l3*-deficient cDC2 cells in cluster 3 have reduced expression of chemokines and cytokines (Mann-Whitney *U* test, *P < 0.05, ***P < 0.001). (**H**) *Dnase1l3*-deficient cDC2 cells in cluster 3 have reduced expression of various activation marker genes (Mann-Whitney *U* test, **P < 0.01, ***P < 0.001, ****P < 0.0001). (**I**) *Dnase1l3*-KO mice have reduced abundance of CD86^+^CD11b^+^ cDCs in the MC38 tumors at an early stage of tumor development (10 days after inoculation) (*n* = 21 WT and 21 KO from 3 independent experiments; *P < 0.05). Representative FACS plots from WT and KO mice are shown. Biological replicates were combined in the analysis using a linear regression model to remove the batch effects (see Methods). (**J**) *Dnase1l3*-KO mice have reduced abundance of activated cDCs in tumor draining lymph nodes (dLNs) at an early stage of tumor development (10 days after inoculation). Indicated immune cell population in dLNs was analyzed by flow cytometry (*n* = 16 WT and 16 KO from 2 independent experiments). Biological replicates were combined in the analysis using a linear regression model to remove the batch effects (see Methods).

To further elucidate how an early-phase activation of proinflammatory responses leads to a late-phase reduction of antitumor immunity in the colon of *Dnase1l3*-KO mice, we established a syngenic colon tumor model by s.c. injecting MC38 murine colon cancer cells into *Dnase1l3* WT and *Dnase1l3-*KO mice. *Dnase1l3*-KO mice developed significantly larger tumors after 18–21 days (Figure 5, A–C, and Supplemental Figure 6), indicating that *Dnase1l3* deficiency in host cells, but not tumor cells, enhances tumor growth. Given that *Dnase1l3* is highly expressed in cDCs (6, 7, 18), these observations further suggest that the transition from the early phase of proinflammatory effect to the late phase of immune-suppressive antitumor state in *Dnase1l3*-KO mice may be mediated by immune cells, particularly cDCs, in the tumor microenvironment.

To test this hypothesis, we carried out single-cell RNA-Seq (scRNA-Seq) analyses of DCs (live CD11c^+^CD26^+^CD45^+^CD88^–^F4/80^–^Ly-6G^–^MHC-II^+^) in MC38 tumors from *Dnase1l3* WT and *Dnase1l3-*KO mice (Supplemental Figure 7A). Clustering analysis of all sequenced DCs identified 14 clusters that could be further functionally annotated into 2 major cDC subtypes, cDC1 and cDC2, and minor contamination of pDCs (Figure 5, D and E, and Supplemental Table 3). cDC1 represented a small fraction among cDCs in the tumors from both WT and *Dnase1l3*-KO mice, and tumors from *Dnase1l3*-KO mice contained an increased proportion of cDC1 compared with those from WT mice (Figure 5, D and E, and Supplemental Table 3; cluster 6, 8, and 13). However, DEG analysis of all intratumoral cDC1 (combined from above 3 clusters) showed that cDC1 from *Dnase1l3*-KO mice displayed only significantly reduced genes compared with those from WT mice (Supplemental Table 4). Further Gene Ontology (GO) and pathway enrichment analyses revealed that these genes were enriched with gene sets associated with antigen processing and presentation as well as protein translation and expression (Supplemental Figure 7B). These observations suggest that cDC1 in the tumors from *Dnase1l3*-KO mice might not be fully functional to activate T cells.

The cluster 3 cDC2 was the most dramatically reduced cDC2 cluster in MC38 tumors from *Dnase1l3*-KO mice (16.75% of total cells from WT compared with 5.8% of total cells from *Dnase1l3*-KO mice) (Figure 5, D and F, and Supplemental Table 3). These cDC2 displayed a unique gene expression profile with low levels of *Itgax* (Cd11c), cDC1 marker *Xcr1*, and cDC2 markers including *Itgam* (Cd11b) and *Sirpa* (*Cd172*) (Supplemental Figure 7C); hence, they could be CD11b^lo^CD172^lo^ but moderate to high levels of chemokines and cytokines, such as *Ccl5*, *Ccr7*, *Cxcl10*, *Ifi205*, and *S100a4* (Figure 5G). These cDC2 resemble a skin-specific cDC subset called double-negative (XCR1^lo^CD11b^lo^) cDC2, although skin CD11b^lo^ cDC2 express *Sirpa* at a high level (27). cDC2 in cluster 3 also expressed high levels of MHC class I genes, such as *H2-D1* and *H2-K1*, and *B2m*-encoding MHC-I–associating molecule b2 microglobulin (Supplemental Figure 7D) as well as MHC class II genes such as *H2-Aa*, *H2-Ab1*, *H2-DMb1*, *H2-Eb1*, and *Cd74* (Supplemental Figure 7E), suggesting their potential role in stimulating CD8^+^ and CD4^+^ T cells. Moreover, gene set variation analysis (GSVA) indicated that the cluster 3 cells displayed the most notable alterations between KO and WT in transcriptional profiles among all clusters (Supplemental Figure 7F). Specifically, cluster 3 cDC2 isolated from MC38 tumors in *Dnase1l3*-KO mice had decreased GSVA scores in gene sets associated with proteasome, graft-host interaction, autoimmune diseases, and antigen processing and presentation, compared with those from WT mice (Supplemental Figure 7F; cDC2, cDC3). The top downregulated genes included chemokines/cytokines *Ccl5*, *Ccr7*, and *Cxcl10* (Figure 5G) and activation markers *Marcksl1*, *H2-DMb2*, *Cd63*, and *Cd86* (Figure 5H). These observations indicate that tumors in *Dnase1l3*-KO mice contain a reduced abundance of a special set of active cDC2 compared with those from WT mice, and these special cDC2 are potentially important to activate antitumor T cells.

Consistent with our observations from scRNA-Seq analysis, when characterizing the infiltrating innate immune cells in the draining lymph nodes (dLNs) and early tumors (10 days after inoculation) from tumor-bearing mice by flow cytometry analyses (Supplemental Figure 8A), we found that tumors isolated from *Dnase1l3*-KO mice had reduced abundance of CD86^+^CD11b^+^ cDC2 compared with tumors in WT mice (Figure 5I). The intratumoral levels of total cDCs and XCR1^+^ cDC1 were comparable between *Dnase1l3*-KO and WT mice (Supplemental Figure 8, B and C). However, relatively few XCR1^+^ cDC1 in dLNs of KO mice expressed the immune activation marker PD-L1 (28), compared with their counterparts in WT mice (Figure 5J). These data suggest that the enhanced tumor growth in *Dnase1l3*-deficient mice might be a result of impaired DC function and consequent reduction in cytotoxic CD8^+^ and CD4^+^ T cells in dLNs and tumors.

### Dnase1l3 deficiency alters cytotoxic T cells in mice and in vitro.

**Figure 6:**
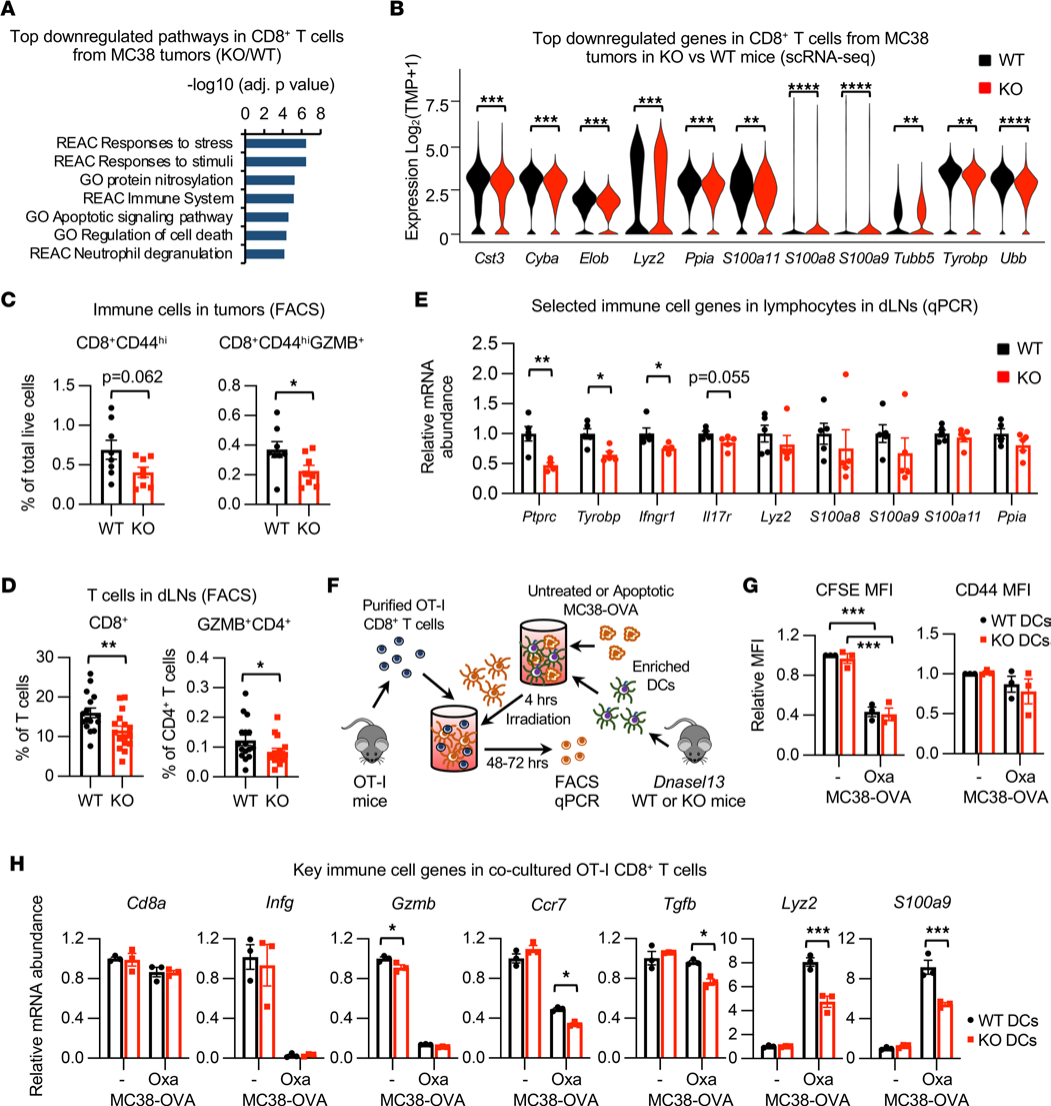
*Dnase1l3* deficiency alters cytotoxic T cells and impairs antitumor immunity. (**A** and **B**) Top downregulated genes (log_2_FC < –0.4) in CD8^+^ T cells from MC38 tumors in *Dnase1l3*-KO mice were analyzed for pathway enrichment (*P_adj_ < 0.05, **P_adj_ < 0.01, ***P_adj_ < <0.001, ****P_adj_ < 0.0001). (**C**) *Dnase1l3*-KO mice have reduced abundance of activated T cells in MC38 tumors 14 days after inoculation. Indicated immune cell populations in isolated tumors were analyzed by flow cytometry (*n* = 8 WT and 8 KO mice, Student’s *t* test, *P < 0.05). (**D**) *Dnase1l3*-KO mice have reduced abundance of activated T cells in tumor draining lymph nodes (dLNs) 10 days after inoculation (analyzed by flow cytometry, *n* = 16 WT and 16 KO from 2 independent experiments; Mann-Whitney *U* test, *P < 0.05, **P < 0.01). (**E**) *Dnase1l3*-KO mice have reduced expression of several immune cell genes in CD45^+^ immune cells isolated from tumor draining lymph nodes (dLNs) 14 days after inoculation (analyzed by qPCR, *n* = 5 WT and 5 KO mice; Mann-Whitney *U* test, *P < 0.05, **P < 0.01). (**F**) Schematic representation of in vitro coculture experiment using OT-I CD8^+^ T cells and WT or *Dnase1l3*-KO DCs in the presence of regular or apoptotic MC38-OVA cells. Apoptosis of MC38-OVA was induced by treatment with 400 μM oxaliplatin (Oxa) for 24 hours. (**G**) OT-I CD8^+^ T cells have comparable proliferation and CD44 activation when cocultured with apoptotic MC38 cells and WT or *Dnase1l3*-KO DCs. The mean fluorescence intensity (MFI) of CFSE dye (for cell proliferation) and the MFI of CD44 in OT-I CD8^+^ T cells were analyzed by FACS (*n* = 3 independent experiments, 2-way ANOVA with correction for multiple comparisons, ***P < 0.001).(**H**) OT-I CD8^+^ T cells coincubated with *Dnase1l3*-KO DCs in the presence of apoptotic MC38 cells have reduced expression of some key immune cell markers (analyze by qPCR, *n* = 3 replicates, 2-way ANOVA test with correction of multiple comparisons, *P < 0.05, ***P < 0.001).

In support of the notion that the enhanced tumor growth in *Dnase1l3*-deficient mice might be a result of impaired DC-mediated activation of cytotoxic T cells, scRNA-Seq analysis of the CD45^+^ cells in MC38 tumors (Supplemental Figure 9A) revealed that tumors from KO mice were enriched with dysregulated cytotoxic T cells compared with those from WT mice (Supplemental Figure 9B and Supplemental Table 4). Among 20 CD45^+^ immune cells clusters, cluster 13 (GZMB^+^CD8^+^ T cells) displayed the biggest reduction in MC38 tumors from *Dnase1l3*-KO mice compared with those from WT mice; 2.63% of total cells were from WT, while 1.57% of total cells were from *Dnase1l3*-KO mice (Supplemental Table 4). In addition to a reduction in cellular abundance, GSVA revealed that the top significantly upregulated gene set in this cluster of GZMB^+^CD8^+^ T cells from MC38 tumors in *Dnase1l3*-KO mice was “Primary immunodeficiency” compared with those from WT mice (Supplemental Figure 9C; CD8, cluster 13), suggesting that they are also dysfunctional. Further DEG analysis of all 3 clusters of CD8^+^ T cells (cluster 7, 12, and 13) showed that, compared with those in WT mice, CD8^+^ T cells from MC38 tumors in *Dnase1l3*-KO mice had a significant reduction in numerous genes involved in regulation of stress response, immune response, protein quality control, and apoptosis/cell death (Figure 6A and Supplemental Table 6). The expression of many genes mediating innate immune function, protein degradation, and programmed cell death — such as lysozyme M *Lyz2*, ubiquitin *Ubb*, and 2 subunits of calprotectin, *S100a8* and *S100a9* (29) — were among the most downregulated genes in CD8^+^ T cells from MC38 tumors in *Dnase1l3*-KO mice (Figure 6B). However, CD8^+^ T cells from MC38 tumors in *Dnase1l3*-KO mice also had significantly higher expression of several genes important in T cell activation, differentiation, and immunodeficiency (Supplemental Figure 9D and Supplemental Table 6).

To validate observations from scRNA-Seq analysis of intratumoral CD45^+^ immune cells, we analyzed infiltrating immune cells in the early tumors and dLNs (10–14 days after inoculation) from tumor-bearing mice by flow cytometry (Supplemental Figure 10A). Consistent with the scRNA-Seq results, MC38 tumors in *Dnase1l3*-KO mice displayed a significant reduction of GZMB^+^CD8^+^CD44^hi^ T cells compared with those from WT mice (Figure 6C), and they displayed with a trend of reduction in total CD45^+^ immune cells and CD8^+^ T cells (Supplemental Figure 10B). In the dLNs, tumor-bearing *Dnase1l3*-KO mice had fewer CD8^+^ T cells and activated GZMB^+^CD4^+^ T cells compared with WT mice (Figure 6D and Supplemental Figure 10C). Subsequent quantitative PCR (qPCR) analysis of CD45^+^ lymphocytes isolated from the dLNs showed that the expression of many upregulated immune genes in intratumoral T cells identified by scRNA-Seq (Supplemental Figure 9D) were significantly reduced or unchanged (but not upregulated) in immune cells from tumor-bearing *Dnase1l3*-KO mice (Figure 6E and Supplemental Figure 10D). Together, these observations indicate that *Dnase1l3* deficiency alters cytotoxic T cells in dLNs and in the tumor microenvironment.

To directly test whether the observed dysregulation of cytotoxic T cells in *Dnase1l3*-KO mice is indeed caused by *Dnase1l3* deficiency in DCs, we performed an in vitro cell culture experiment in which DCs isolated from WT or *Dnase1l3-*KO mice were cocultured with untreated or oxaliplatin-treated (Oxa-treated) apoptotic MC38 cells overexpressing ovalbumin (MC38-OVA). Apoptotic MC38-OVA cells were tested because DNASE1L3 is known to digest DNA encapsulated in microparticles released from cells upon stress or death (5, 6). Naive CD8^+^ T cells isolated from OT-I mice, which can specifically recognize OVA expressed by MC38-OVA cells, were then added to these MC38-OVA “elicited” WT or *Dnase1l3-*KO DCs to analyze their impacts on the proliferation and activation of naive OT-I CD8^+^ T cells (Figure 6F). Both WT and *Dnase1l3-*KO DCs cocultured with apoptotic (Oxa) MC38-OVA had an enhanced ability to promote the proliferation of OT-I CD8^+^ T cells compared with those cocultured with untreated MC38-OVA, as revealed by the marked reduction of the CFSE fluorescence intensities of OT-I CD8^+^ T cells (Figure 6G, left). The ability of *Dnase1l3-*KO DCs to induce the expression of activation marker CD44 was also comparable with that of WT DCs in both conditions (Figure 6G, right). Further qPCR analysis revealed that *Dnase1l3-*KO DCs cocultured with apoptotic MC38-OVA exhibited a reduced ability to alter the expression of several genes involved in activation and/or modulation of T cells (Figure 6H). In particular, the mRNA levels of *Lyz2* and *S100a9*, 2 top downregulated genes in CD8^+^ T cells from syngenic MC38 tumor in KO mice (Figure 6A and Supplemental Table 6), were dramatically induced in OT-I CD8^+^ T cells cocultured with WT DCs loaded with the apoptotic MC38-OVA (Figure 6H). This induction was significantly blunted in OT-I CD8^+^ T cells cocultured with KO DCs loaded with the apoptotic MC38-OVA (Figure 6H). Given the importance of calprotectin (S100a8 and S100a9 heterodimer) in induction of autophagy and apoptosis (29, 30), this observation suggests that *Dnase1l3* deficiency in DCs could directly affect the tumor-killing ability of cytotoxic T cells. Collectively, our analyses indicate that tumors in *Dnase1l3*-KO mice have fewer active CD11b^lo^CD172^lo^ cDC2 compared with *Dnase1l3* WT mice, and this deficiency is associated with dysregulation of cytotoxic T cells in *Dnase1l3*-KO tumors. These immune defects in *Dnase1l3*-KO mice and DCs may directly contribute to impaired antitumor immunity, resulting in increased tumor growth and progression.

## Discussion

Despite a well-established causal relationship between *Dnase1l3* deficiency and autoimmune diseases (6, 7, 11, 17, 31, 32), little is known about the link between *Dnase1l3* deficiency, antitumor immunity, and tumorigenesis/tumor progression. In the present study, we identified the reduced expression of *DNASE1L3* as a potential biomarker for poor prognosis and reduced tumor immune infiltration in multiple human cancers through bioinformatics analyses of TCGA data sets (Figure 1 and Supplemental Figure 1), and this observation was recently confirmed by another bioinformatics study performed in CRC (33). We went beyond these observed associations and provided the first genetic evidence to our knowledge for the tumor-suppressor role of DNASE1L3 using *Dnase1l3*-KO mice. Importantly, in contrast to recent studies that proposed that DNASE1L3 suppresses tumor growth via cancer cell–autonomous mechanisms such as increasing apoptosis, modulating glycolysis, or decreasing migration using epithelial cancer cells with artificially overexpressed DNASE1L3 (33, 34), we demonstrate that *Dnase1l3* deficiency in the tumor microenvironment rather than in tumor cells is sufficient to induce tumor growth and impair antitumor immunity. Specifically, we show that downregulation of *DNASE1L3* in cancer, observed by others and in the present study, stems from specific downregulation of this gene in DCs (Figure 1C and Supplemental Figure 1C), the main cell type that physiologically expresses *DNASE1L3*. Moreover, in the AOM/DSS CRC model, *Dnase1l3* deficiency exacerbated early-phase inflammation that was associated with diminished type I IFN response, and this further led to reduced T cell accumulation and enhanced tumor formation at the later stage (Figure 3 and Supplemental Figure 5). Our syngenic MC38 tumor experiments, performed in immunocompetent *Dnase1l3*-KO mice, further demonstrated that deletion of *Dnase1l3* in the recipient microenvironment rather than in the s.c. grafted epithelial tumor cells, promotes tumor growth, decreases activation of cDC1 and cDC2, and diminishes the abundance and function of cytotoxic T cells in tumors (Figures 5 and 6 and Supplemental Figure 6).

Intriguingly, in a MC38 syngenic tumor model, a unique CD11b^lo^CD172^lo^ subset of cDC2 (cluster 3 in Figure 6, A and C) was profoundly reduced in the tumors of *Dnase1l3*-deficient mice. cDC1 are known to activate cytotoxic CD8^+^ T cells by their superior ability of cross-presentation, but cDC2 are also able to activate CD8^+^ T cells in addition to CD4^+^ T cells (35). It has been recently reported that CD11b^lo^CD172^+^ cDC2 in normal skin stimulates differentiation of type 2 Th cells (27). Our study suggests that CD11b^lo^CD172^lo^ cDC2 in tumors might play important roles for activation of CD4^+^ and CD8^+^ cytotoxic T cells, and reduction of those cDCs might be reflected by tumor progression in *Dnase1l3*-deficient mice. It will be of great interest to further investigate the role of DNASE1L3 on cDC function in future studies.

Monocytes and macrophages play an important role in control of tumor growth and progression. The expression of markers of monocytes/macrophages were induced in tumors in both WT and *Dnase1l3*-deficient mice in the AOM/DSS CRC model (Figure 4C), but proinflammatory cytokines produced by those cells were not sufficient to suppress tumor progression in *Dnase1l3*-KO mice (Figure 3). GSVA of all genes from scRNA-Seq analysis of CD45^+^ immune cells in MC38 tumors (Supplemental Figure 9C, Mono) suggests that this is possibly due to the functional impairment of these cells after inactivation of *Dnase1l3*. Specifically, among all sequenced cell clusters, monocytes from tumors in *Dnase1l3*-KO mice displayed the most significant alterations at the transcriptional level and had decreased GSVA scores in gene sets associated with pathways involved in drug metabolism and DNA repair and recombination (Supplemental Figure 9C, Mono). Intriguingly, these cells also had transcriptional upregulation of genes involved in antigen processing and presentation as well as calcium signaling and membrane potential (LTP) (Supplemental Figure 9C, Mono). Further understanding of DNASE1L3-mediated regulation of monocytes/macrophages will help to elucidate the full picture of the role played by this unique DNASE in antitumor immunity.

Our study has several important clinical implications. First, the antitumor role of DNASE1L3 in CRC development uncovered in our study has implications in inflammatory bowel disease (IBD) or colon cancer treatment in the clinic; the antitumor role of DNASE1L3 suggests that DNASE1L3 protein or a *DNASE1L3*-carrying vector may be considered as a potential therapeutic tool to help colon recovery or improve colon cancer therapy. Second, our bioinformatic analysis of cancer patients indicates that DNASE1L3 could be evaluated as a predictive biomarker for risk and prognosis of various human cancers, and this is also supported by 2 recent studies (33, 36).

In summary, our study uncovers direct involvement of DNASE1L3 in promoting tissue recovery after DNA damages and activation of cDCs and cytotoxic CD8^+^ T cells in the tumor microenvironment and highlights the importance of this enzyme in activation of antitumor immunity and suppression of tumor progression. Our findings further suggest that restoration of DNASE1L3 activity may represent a potential therapeutic approach for anticancer therapy.

## Methods

Supplemental Methods are available online with this article.

### Animal experiments

All mice were maintained at the NIEHS animal facility under strict specific pathogen–free conditions and housed in microisolator static cages (Techniplast) with autoclaved nesting material (Nestlet; Ancare Corp.) and on hardwood bedding (Sani-Chips; P.J. Murphy).

For the chronic inflammation-related colon cancer model (AOM/DSS-colon cancer model), 3-month-old *Dnase1l3* WT (*Dnase1l3^+/+^)* and KO (*Dnase1l3^–/–^)* mice on the C57BL/6 background were injected with AOM (8 mg/kg, body weight). One week later, mice were challenged with 2.5% DSS water for 7 days, followed by a 14-day recovery with regular drinking water for 3 cycles. Body weight, rectal bleeding, and diarrhea were monitored during the entire experiment. Mice with more than 25% weight loss were removed during the experiment.

Additional humane endpoints for the removal of experimental animals on AOM/DSS model include the following. Any 3 of the clinical signs in Category A or any 1 clinical sign in category B will result in the immediate euthanasia of that animal. Category A includes ruffled hair coat, hunched posture, lethargy, weight loss of 20%, rectal prolapse greater than 2 mm, pale extremities, and bloating. Category B includes weight loss of greater than or equal to 25% that does not improve or stabilize in 24 hours of close observation, inability to move about the cage, inability to right itself, labored breathing, rectal prolapse greater than 5 mm, and rectal bleeding score of 3. Diarrhea, mild rectal prolapse (2 mm or less), and mild rectal bleeding (score of 1 or 2) were expected with this model and were not used as criteria for removal.

We also worked with NIEHS veterinarian medicine staffs (VMS) and established appropriate supplemental care for the close monitoring of the procedures and animals. For example, during DSS-treatment, some mice were given food mash (NIH-31 from Harlan Laboratories) prepared with DSS-water. During the water rest period, which was a 14-day recovery period during which mice were given regular drinking water, mice were given normal food mash every day.

For the AOM model, 2-month-old *Dnase1l3* WT and *Dnase1l3-*KO mice were injected i.p. with AOM (8 mg/kg, body weight). Colon tissues were isolated 9 hours or 5 days after injection.

For the doxorubicin model, 2-month-old *Dnase1l3* WT and KO mice were injected i.p. with doxorubicin (10 mg/kg, body weight). Intestine was isolated 12 hours, 3 days, or 5 days after the injection.

For the syngenic MC38 cancer model, we performed analysis at 2 time points — early and late. For the late time point experiment, 8- to 12-week-old *Dnase1l3* WT and *Dnase1l3-*KO mice were injected s.c. with 1.0 × 10^5^ MC38 cells into each flank. Mice were monitored twice weekly for 21 days. Tumor length and width were measured by caliper, and tumor volume was calculated using the formula V = length × width^2^/2. Mice were sacrificed at day 21 after the injection of the tumor cells or when their total tumor volume reached the volume of 2,000 mm^3^. The experiments were independently performed 5 times in both male and female mice by 2 different researchers during this study, and consistent results were obtained. For the early time point, 8- to 12-week-old female WT and KO mice were the injected s.c. with 2.5 to 5.0 × 10^5^ MC38 cells into the back. Mice were monitored twice weekly for tumor growth and health status. Mice were sacrificed at day 10 or 14 for the analysis of the immune cell populations in tumors and tumor draining lymph nodes.

The group size in each experiment was designed to provide 80% or 90% power (dependent on different experimental outcomes in different mouse strains) to detect the expected magnitude of changes. Animals were randomly allocated into experimental groups to minimize the potential litter/cage effect. During each procedure, WT and KO animals were also treated and harvested in an alternating order to control possible confounding issues. The researchers were not blinded to animal genotypes and treatments during all stages of experiments and data analysis.

### RNA extraction and qPCR

RNA was extracted from tissue using RNeasy Mini kit (Qiagen) according to manufacturer protocol. In total, 1 mg RNA was reverse transcribed using High-Capacity cDNA Reverse Transcription Kit (Thermo Fisher Scientific). qPCR was performed using iQ SYBR Green Supermix (Bio-Rad) on CFX96 real-time PCR instrument (Bio-Rad). Relative mRNA levels were normalized with Lamin A. Fold difference was determined by 2^–ΔΔCT^ method. Sequences of qPCR primers can be found in Supplemental Table 7.

### RNA-Seq analysis of colon tissues and colorectal tumors in mice under the AOM/DSS procedure

RNA was extracted from the whole colon tissue of *Dnase1l3* WT and *Dnase1l3-*KO mice at basal condition (D0), after 1 cycle of AOM/DSS (D19), after 3 cycles of AOM/DSS (D80), and from final tumors from *Dnase1l3* WT and *Dnase1l3-*KO mice during the AOM/DSS procedure. For each group, 3–5 samples were used. Comparisons were carried out between the 2 genotypes for each group. RNA was extracted using RNeasy mini kit (QIAGEN) and was of sufficient quality. Single-ended (75 bp) sequencing was carried out in house using Illumina NextSeq 500 to at least 30 million reads per sample. The quality of reads was evaluated using FastQC (v0.11.5). Reads were aligned using STAR (version 020201) (37). The STAR index was built using mouse genome (mm10, GENCODE release vM25). Transcript expression was quantified using the subread package (38) (version 1.6.2) to generate raw counts for 55,293 genes. Lastly, a variance-stabilizing transformation was applied to raw counts from all genes using the DESeq2 (39) software (version 1.30.0) in R to identify DEGs, assuming a negative binomial distribution for count values. A gene was considered differentially expressed when its adjusted *P* < 0.05 and fold change > 2.0. For tumor samples, DEGs were declared with nominal *P* < 0.01 (without adjusting for multiple comparisons) and fold change > 2.0. Gene set enrichment analysis (GSEA) was carried out using DAVID (40) and gProfiler (https://biit.cs.ut.ee/gprofiler/gost).

### Flow cytometric analysis and sorting

Cells were diluted to 0.5 × 10^6^/100 μL to 1 × 10^6^/100 μL and incubated with a nonspecific binding blocking reagent cocktail of anti–mouse CD16/CD32 antibody (2.4G2) (10% culture supernatant), 5% normal mouse, and 5% rat serum (Jackson ImmunoResearch) (41). For DC analysis, cell surface antigens were stained with following fluorochrome-conjugated antibodies obtained from BD Biosciences (BD), BioLegend (BL), or eBioscience/Thermo Fisher Scientific (eBio) on ice for 30 minutes. Biotin anti–mouse CD3e (145-2C11, BD, 553060), BUV395 anti–mouse CD11b (M1/70, BD, 563553), PerCP-Cy5.5 anti–mouse CD11c (N418, eBio, 45-0114-82), biotin anti–mouse CD19 (6D5, BL, 115504), BV510 anti–mouse CD45.2 (104, BL, 109867), FITC anti–mouse CD86 (GL1, BD, 561962), PE anti–mouse CD88 (20/70, BL, 135806), eFluor450 anti–mouse MHC class II I-Ab (AF6-120.1, eBio, 48-5320-82), BV711 anti–mouse PD-L1 (10F.9G2, BL, 124319), and Alexa Fluor 647 anti–mouse XCR1 (ZET, BL, 148214). Biotinylated antibodies were followed by PE-Dazzle594–conjugated streptavidin (BL, 405247). Stained cells were fixed by 1% paraformaldehyde. For T cell analysis, cell surface antigens were stained with following fluorochrome-conjugated antibodies: BUV395 anti–mouse CD4 (RM4-5, BD, 740208), BV510 anti–mouse CD8α (53-6.7, BD, 563030), biotin anti–mouse CD44 (1M7, BD, 553132), APC anti–mouse CD62L (MEL-14, eBio, 17-0621-81), BV711 anti–mouse PD-1 (29F.1A12, BL, 135231), PE anti–mouse TCR Cβ (H57-597, BD, 553172), APC anti–mouse CD62L (MEL-14, eBio, 17-0621-81), BV711 rat IgG2a (RTK2758, BL, 400551), and FITC rat IgG2a (R35-95, BD, 554688). Dead cells stained with eFluor780-conjugated Live/Dead dye (eBio, 65-0865-18). Biotinylated antibodies were followed by PerCP-Cy5.5–conjugated streptavidin (BD, 551419). For staining intracellular proteins in T cells, cells were fixed and permeabilized using a kit (eBio, 00-5521) according to manufacturer instruction then stained with eFluor450 anti–mouse Foxp3 (FJK-16s, eBio, 48-5773-80) and FITC anti–mouse granzyme B (GZMB) (QA16A02, BL, 372205). Stained cells were analyzed on LSR-Fortessa flow cytometer (BD), and the data were analyzed using FACS Diva (BD) and FlowJo (Tree Star Inc.) software. Only single cells were analyzed, and dead cells were excluded from analysis. Gating strategies used for differentiating the immune cell populations are shown in Supplemental Table 8, and all antibodies used in this study can be found in Supplemental Table 9.

### Single-cell RNA-Seq (scRNA-Seq) analysis of tumor-associated DCs and CD45+ immune cells

#### Isolation of DCs and T cells for scRNA-Seq analysis.

MC38 tumor cell line was s.c. injected into mouse flanks (5 × 10^5^ each side), and tumors were excised from euthanized mice without skin, lymph nodes, or connective tissues 10 days after the injection. Minced tumors were digested with Liberase TM (100 μg/mL) (Roche), Collagenase XI (250 μg/mL), Hyaluronidase (1 mg/mL), and DNase I (200 μg/mL) (Sigma-Aldrich) for 30 minutes at 37°C (41). The reaction was stopped by the addition of EDTA (20 mM final concentration). A single-cell suspension was prepared by sieving the digested tissue through a 70 μm nylon strainer (BD Biosciences). DCs and T cells were enriched by double-gradient centrifugation at 455*g* for 20 minutes at room temperature using 14.5% Nycodenz (Accurate Chemical) (low density for DCs) and Histopaque 1083 (MilliporeSigma) (high density for T cells). Cells were collected from each interface and then washed with PBS containing 0.5% BSA (Sigma-Aldrich) and 2 mM EDTA (Sigma-Aldrich).

#### scRNA-Seq of tumor-associated DCs and CD45^+^ immune cells.

Tumor-associated DCs and total leukocytes were isolated and enriched as described above. Cell surface antigens were stained with following fluorochrome-conjugated antibodies together with eFluor780-conjugated Live/Dead dye (Thermo Fisher Scientific). PerCP-Cy5.5 anti–mouse CD11c (N418, eBio, 45-0114-82), APC anti–mouse CD26 (H194-112, BL, 137807), BV711 anti–mouse CD45.2 (104, BL, 137807), PE anti–mouse CD88 (20/70, BL, 135806), PE-Dazzle594 anti–mouse F4/80 (BM8, BL, 123146), eFluor450 anti–mouse MHC class-II I-Ab (AF6-120.1, eBio, 48-5320-82), and BV510 anti–mouse Ly-6G (1A8, BL, 127633) were used. Among stained cells, cDCs (CD11c^+^CD26^+^CD45.2^+^CD88^–^F4/80^–^I-A^+^Ly-6G^–^Live/Dead^–^) or leukocytes (CD45.2^+^Live/Dead^–^) were purified using a cell sorter FACS ARIA-II (BD Biosciences). The cells were counted and examined for viability using a TC-20 cell counter (Bio-Rad). About 8,000–9,900 live cells at 2 × 10^5^ cells/mL concentration with 70% or higher viability were loaded into the Single Cell Ship, and were then followed by formation of single-cell emulsion in Chromium Single Cell Controller using Chromium Single Cell 3′ Library & Gel Bead Kit v3.1 (10x Genomics). The mRNA reverse transcription, cDNA generation and amplification, and single-cell gene expression library construction were carried out according to the protocols provided by the manufacture. The libraries were then sequenced by the NIEHS Epigenomics and DNA Sequencing Core Laboratory on NovaSeq 6000 (Illumina) with paired-end sequencing (Read 1: 28 bases; Read 2: 90 bases). In total, 8.7 × 10^8^ reads were obtained for the 4 samples.

#### scRNA-Seq data processing.

Raw sequencing data were demultiplexed, aligned, and counted with Cell Ranger pipelines (10x Genomics). First, we used cellranger *mkfastq* command to generate FASTQ files. Then, we extracted expression data at a single-cell resolution using cellranger command *count*. Lastly, we used cellranger *aggr* to combine sequencing data from multiple libraries with mapped sequencing depth. We obtained 4 expression files for cDC and CD45^+^ populations isolated from *Dnase1l3*-KO and WT mice.

#### Clustering of scRNA-Seq data.

R package Seurat 4.0 (42) was used for gene and cell filtering, expression normalization, principal component analysis (PCA), variable gene finding, clustering analysis, and UMAP analysis. Analyses were performed with default parameters unless specified otherwise. First, the 4 gene-by-cell expression data matrices from the aggregated library were imported separately to create 4 Seurat objects. Based on visual inspection, we filtered out cells using the following criteria: (a) cells expressed < 200 or > 5,000 genes; (b) cell counts were > 25,000; and (c) proportions of mitochondrial gene expression in cells were larger than 10%. Data were then log-transformed (log_2_[transcripts per million (TPM)+1]) for subsequent analysis. PCA was performed for dimension reduction using the top 2,000 most variable features. We then did a cell cycle analysis to further filter out variations that are not related to the underlying biology. We acquired both S phase genes and G2/M phase genes and assigned cell cycle scores by using the *CellCycleScoring* function. We subtracted (regressed out) this source of heterogeneity from the data by using the *ScaleData* function with variables to regress using both calculated S and G2/M cell cycle scores. To combine WT and KO data sets, we first selected features that were repeatedly variable across WT and KO data sets for cDCs and CD45^+^ cells separately, using the *SelectIntegrationFeatures* function. We then identified anchors using the *FindIntegrationAnchors* function, which takes both WT and KO Seurat objects as input, and we used these anchors to integrate the 2 data sets with *IntegrateData* function. We ran the standard workflow for the visualization and clustering as follows: We (a) used *ScaleData* function on the integrated data; (b) performed PCA using the *RunPCA* function with 30 PCs; (c) used *RunUMAP* with PCA reduction with the first 30 PCs; (d) calculated nearest neighbors by using the *FindNeighbors* function with the selected 30 PCs; and (e) identified clusters for each data set by using the *FindClusters* function with resolution of 0.5. For cluster analysis, *FindNeighbors* function with the first 10 principal components was used for clustering analysis. We also performed differential gene expression analyses using 2-tailed Student’s *t* test (*t.test* function in R) between the WT and KO cells in each cluster. We then adjusted for multiple testing by the FDR method (*p.adjust* function with ‘*fdr’* in R). Marker gene expression levels in different conditions were visualized with the R package *ggplot2* violin plots.

#### GSVA of scRNA-Seq data sets.

GSVA was performed using the GSVA R package (43) in R3.6.1 on gene expression counts from tumor-associated DCs or CD45^+^ immune cells isolated from WT and *Dnase1l3^–/–^* mice without background genes. Gene sets were sourced from MSigDB 7.2 (www.gsea-msigdb.org) and TRANSFAC 7.0. We used output from GSVA to fit a linear model using the “lmFit” function and used the “eBayes” function from the limma R package (44) to compute moderated t-statistics, moderated F-statistic, and log-odds of differential expression by empirical Bayes moderation. Resulting *P* values were corrected by Benjamini-Hochberg (BH) for multiple comparisons.

### In vitro OT-I CD8+ T cell proliferation and activation induced by MC38-OVA loaded WT or Dnase1l3-KO DCs

MC38-OVA were cultured in regular complete DMEM and were then treated with or without 400 μM Oxa for 24 hours to induce apoptosis. Spleens from WT or *Dnase1l3*-KO mice were digested, and splenocytes were purified by gradient centrifugation (455*g* for 20 minutes at room temperature) with 14.5% Nycodenz. WT or *Dnase1l3*-KO DCs were then enriched from purified splenocytes using MACS bead–conjugated anti-CD11c (Miltenyi Biotec) and AutoMACS (program possel-s; Miltenyi Biotec). Oxa-treated apoptotic MC38-OVA cells and untreated MC38-OVA cells (1 × 10^6^) were washed and mixed with 1 × 10^6^ WT or *Dnase1l3*-KO DCs and incubated for 4 hours; then, MC38-OVA and DC culture were irradiated with 30 Gy γ-ray to stop the growth of MC38-OVA cells and to minimize DC numbers in the subsequent T cell analysis. DCs were washed and resuspended in cRPMI-10 medium at 5 × 10^5^/mL.

Naive CD8^+^ T cells were purified from skin-draining LNs, mesenteric LNs (MLNs), and spleens from naive OT-I mice (The Jackson Laboratory, 003831) using antibody cocktail (anti-CD4, -CD11b, -CD11c, -CD16/32, -CD19, -CD25, -CD44, -B220, -CD49b, -Ly6C/G, –I-Ab, and -TER119) and AutoMACS (program depl 025). Naive CD8^+^ T cells were then labeled with 3 μM CFSE (Thermo Fisher Scientific) at 37°C for 15 minutes and then resuspend in cRPMI-10 at 1 × 10^6^/mL. Finally, 1 × 10^5^ CFSE-labeled OT-I CD8^+^ T cells (100 μL) and 5 × 10^4^ DCs cocultured with apoptotic MC38-OVA from above steps (100 μL) were added into a 96-well U-bottom plate, incubated at 37°C for 48 hours. Final coincubated OT-I CD8^+^ T cells were stained with CD44 antibody and Live/Dead dye and were analyzed by flow cytometry, or they were directly subjected to qPCR analysis.

### Statistics

Values are expressed as mean ± SEM from at least 3 independent experiments or biological replicates. Significant differences between the means with 2 comparison groups were analyzed by the 2-tailed, unpaired, nonparametric Mann-Whitney *U* test (sample size < 4) or 2-tailed Student’s *t* test (sample size < 4) (45). Significant differences between the means with more than 2 comparison groups were analyzed by 2-way ANOVA, with adjustments for multiple comparisons using Tukey’s HSD. For all comparisons, differences were considered significant at *P* < 0.05. For qPCR analysis in Figure 4C, outliers that fell below Q1 − 3.0 × IQR or above Q3 + 3.0 × IQR were removed. No samples were excluded from other data analyses.

Data processing and analysis of RNA-Seq and scRNA-Seq data were described in above. Software and algorithms used in this study for data analysis are included in Supplemental Table 10.

### Study approval

All animal experiments were designed and conducted in accordance with guidelines of NIEHS/NIH Animal Care and Use Committee under the Animal Study Proposal no. ASP2018-0018.

### Data availability

The Gene Expression Omnibus (GEO) accession no. for the bulk RNA-Seq data set generated with colonic tissues or tumors from WT and *Dnase1l3*-KO mice under the AOM/DSS procedure at different time points is GSE196077. The GEO accession no. for scRNA-Seq data sets generated with tumor-associated DCs or CD45^+^ immune cells from MC38 tumors on WT and *Danse1l3*-KO mice is GSE196054. The publicly available TCGA database used in this study is available from https://gdac.broadinstitute.org/ Additional information about DEGs and gene clusters in the bulk RNA-Seq data set is included in Supplemental Tables 1 and 2. The scRNA-Seq results on immune cell clusters and DEGs are in Supplemental Tables 3–6. All oligos used in the study are listed in Supplemental Table 7. The gating strategies for flow cytometric analysis of the immune cell populations are listed in Supplemental Table 8. All antibodies used in the study are listed in Supplemental Table 9. Software and algorithms used in this study for data analysis are included in Supplemental Table 10. The raw data of all figures are included in the Supporting Data Values file. Unedited immunoblots for Figure 4D are provided as a supplementary file (Full unedited gel for Figure 4D).

## Author contributions

LL conceived the project and designed the study. WL carried out most of the experiments, with help from PS, WF, FZ, and PWK. HN and KN performed the scRNA-Seq, the flow cytometry analysis, and the in vitro coculture experimentm with help from XX and WF. YL, MS, LL, WL, and SAG performed data analysis. XL, IS, DNC, MBF, and YW guided, designed, and coordinated the study. DK and RM provided *Dnase1l3*-KO mice. WL, XL, and LL wrote the manuscript, with input from all coauthors.

## Supplementary Material

Supplemental data

Supplemental table 1

Supplemental table 10

Supplemental table 2

Supplemental table 3

Supplemental table 4

Supplemental table 5

Supplemental table 6

Supplemental table 7

Supplemental table 8

Supplemental table 9

Supporting data values

## Version 1 08/15/2023

In-Press Preview

## Version 2 09/08/2023

Electronic publication
