# Supplementary material for: DNASE1L3 enhances antitumor immunity and suppresses tumor progression in colon cancer
Source: JCI Insight. 2023 Sep 8;8(17):e168161. doi: 10.1172/jci.insight.168161 (PMC10544201; doi:10.1172/jci.insight.168161)
Supplement: Supplemental data [file jciinsight-8-168161-s207.pdf]

Supplementary Methods

Cell lines

MC38 cells were gifted by Dr. Jeffrey Schlom from NCI of NIH (Bethesda, MD) and by Dr. Andrew Y. Koh from University of Texas Southwestern Medical Center (Dallas, TX). MC38-OVA cells were created by transducing MC38 cells with lentiviral particles containing the OVA-miSFIT-17-T construct (Addgene #124677, a gift from Tudor Fulga) (1). Stable GFP-positive MC38-OVA cells were sorted out by FACS 5 days after transduction. The cells were cultured with complete DMEM media supplemented with 10% heat-inactivated fetal bovine serum (HI-FBS) (Hyclone, Cytiva, Global Life Sciences Solutions USA LLC., Marlborough, MA, USA), 1% penicillin-streptomycin (Pen-strep) (Life Technologies Corporation, Waltham, Massachusetts, USA). The media was changed every 2 days. The cells were harvested by trypsinization and washed twice with endotoxin-free PBS. The cells were tested negative for mycoplasma contamination on periodic quality control check.

Generation of the *Dnase1l3$^{+/+}$;APC$^{min/+}$* and *Dnase1l3$^{-/-}$;APC$^{min/+}$* mice

All mice were on *C57BL/6J* background. *Dnase1l3$^{-/-}$;APC$^{min/+}$* and their age- and gender-matched *Dnase1l3$^{+/+}$;APC$^{min/+}$* controls were generated by crossing *Dnase1l3$^{-/-}$* mice with *APC$^{min/+}$* mice and *Dnase1l3$^{+/+}$* mice with *APC$^{min/+}$* mice, respectively. Age- and gender matched littermates were used for all experiments except stated otherwise.

Histological evaluation of colon tissues from mice treated with AOM/DSS

Tumors formed in the colon of AOM/DSS-treated mice were counted, and tumor burden was calculated as ratio between tumor area and total colon area. The total tumor area in one animal was calculated by summing up the areas of all tumors from that mouse.

Hematoxylin and eosin (H&E) stained Swiss roll sections of large intestine (colon) were blindly evaluated by a certified pathologist for the presence of tumors, their stages, as well as proliferative and non-proliferative lesions (inflammation and ulcer). Lesion descriptions, morphologic diagnoses, and severities are provided as follows. **Atypical hyperplasia** was characterized by crypts lined with crowded epithelial cells that still maintained polarity, with some dysplasia. This lesion was smaller in size and less proliferative than one would expect with an adenoma or adenocarcinoma. **Adenomas** were either composed of proliferations of branching tubules in the lamina propria or finger-like projections of lamina propria lined with proliferating epithelium. There were varying degrees of dysplasia evident in the adenomas; the epithelial cells typically lacked their normal layering and organization, with cells piling upon each other. Nuclei varied in shape and size, but they were often large and basophilic. **Adenocarcinoma** was a sessile proliferation of epithelial cells, with invasion into the underlying lamina propria and submucosa.

Immunohistochemistry assays

Immunohistochemical stained colonic tissues from *Dnase1l3* Wt and KO mice treated with AOM/DSS were formalin-fixed, paraffin-embedded, and sectioned to slides. The slides were deparaffinized in xylene and rehydrated through graded ethanol. Endogenous peroxidase was blocked using 3% H_2O_2, after which heat-induced epitope retrieval was performed using a 1 x EDTA buffer solution (Biocare Medical, Concord, CA) in a Decloaker pressure chamber for 5 min at 120℃. Non-specific sites were blocked with R.T.U. normal goat serum 2.5% (ImmPRESS Reagent Kit Anti-Rat Ig (peroxidase) (Vector Laboratories, Inc., Burlingame, CA) for 20 min at room temperature. Sections were incubated with rat anti-mouse CD8a monoclonal antibody (4SM15) (invitrogen, Catalog # 14-0808-82; 1:500 dilute) for one hour at room temperature. Rat IgG2a isotype control serum was applied to the negative control. Further, the sections were incubated with ImmPRESS Reagent Kit Anti-Rat Ig (peroxidase) (Vector Laboratories, Inc.,

Burlingame, CA) for 30 min at room temperature. The antigen antibody complex was visualized using 3-diaminobenzidine (DAB) chromagen (Dakocytomation, Carpenteria, CA) for 6 min at room temperature. Finally, the sections were counterstained with hematoxylin, dehydrated through graded ethanol, cleared in xylene, and cover slipped. The percentage of CD8a positive area was calculated as $CD8a^+$ area/total tumor area using the ImageJ 2.0.0 (Fiji) software. Tumor sections from each mouse were blinded quantified, and the results were averaged for each mouse.

For CD3 staining: Formalin-fixed paraffin-embedded mouse intestine tissues were deparaffinized and rehydrated through graded ethanol. Heat-induced epitope retrieval was performed using a citrate buffer solution, pH 6.0 (Biocare Medical, Concord, CA, Rodent Decloaker, Cat # RD913M) in the Decloaking pressure chamber for 15 minutes at 110°C. Endogenous peroxidase was quenched using 3% H_2O_2, after which non-specific binding was blocked using Rodent Block M (Biocare Medical, Concord, CA) for 20 minutes at room temperature. The sections were then incubated with rabbit polyclonal anti-CD3 antibody (Abcam, Cambridge, MA, Catalog # ab5690, Lot # GR317048-1) at a 1:750 dilution for one hour at room temperature. Negatives were stained with normal rabbit IgG control serum (Calbiochem, Cat # NI01, Lot # 2659621) at a 1:375 dilution to match the protein concentration of the primary antibody. For detection, the slides were incubated in Rabbit-on-Rodent HRP-Polymer Detection (Biocare Medical, Concord, CA, Catalog # RMR622) for 30 minutes at room temperature. The antigen-antibody complex was visualized with 3,3'-diaminobenzidine (DAB) chromogen (Dakocytomation, Carpinteria, CA) for 6 minutes. Finally, the sections were counterstained with hematoxylin, dehydrated through graded ethanol, cleared in xylene, and coverslipped. Percentage of CD3 positive area were quantified and calculated similar as CD8.

TUNEL staining

TUNEL staining of formalin-fixed, paraffin-embedded mouse colon tissue samples was performed using the manufacturer's recommendations contained within the ApopTag Plus Peroxidase *in situ* Apoptosis Detection Kit (Cat# S7101, Millipore, Billerica, MA). Enzyme-induced epitope retrieval was performed using a proteinase K (Cat# S3020, DakoCytomation, Carpenteria, CA) for 90 seconds at RT, after which endogenous peroxidase blocking was done by immersing the sections in 3% H_2O_2 for 15 min. TdT enzyme incubation was performed for 15 min at 37°C at a dilution of 1:5 (11 µl dH_2O, 77 µl Reaction buffer, 22 µl TdT per 110 µl). Staining was visualized using 3-diaminobenzidine (DAB) chromogen (DakoCytomation, Carpenteria, CA) and counterstained with hematoxylin. Slides were dehydrated through graded ethanol, cleared in xylene, and cover slipped.

Genomic DNA and nuclear-DNA degradation assay

Genomic DNA degradation assay was performed as described (2). In brief, purified genomic DNA was isolated by DNeasy blood & tissue kits (Qiagen, 69504). 5 µl of serum from either *Dnase1l3* WT or KO mice was added to 50 µl of substrate solution (500 ng genomic DNA in 25 mM Tris-HCl (pH 7.4), 150 mM KCl, 5 mM $MgCl_2$, 5 mM $CaCl_2$) and incubated at 37°C at the indicated times. For nucleosome DNA degradation assay, nuclei were isolated as described (3). In brief, 1 x 10^6 cells were suspended in 1 mL of cold isolation buffer (10 mM KCl, 250 mM sucrose, 4 mM $MgCl_2$, 1 mM dithiothreitol, 20 mM Hepes) with 10 µL of protease inhibitor (Thermo Fisher Scientific, Waltham, MA, USA). Then, the cells were lysed with the addition of 50 µl of 0.5% NP-40. After 10-min incubation on ice, cell nuclei were harvested by centrifugation (200 g x 5 min). Precipitated nuclei were rinsed with 1 mL of cold isolation buffer twice and incubated with 15 µl serum in 100 µl solution buffer (10 mM Hepes / 50 mM NaCl / 2 mM $MgCl_2$ / 2 mM $CaCl_2$ / 40 mM β-glycerophosphate, pH 7.0) at 37°C for 2 h.

Reagents

Azoxymethane (Sigma, A5486), DSS Dextran sulfate sodium salt (Sigma, 42867-100G), doxorubicin hydrochloride (Sigma, D1515-10MG).

References:

1. Michaels YS, Barnkob MB, Barbosa H, Baeumler TA, Thompson MK, Andre V, et al. Precise tuning of gene expression levels in mammalian cells. *Nat Commun.* 2019;10(1):818.
2. Mizuta R, Araki S, Furukawa M, Furukawa Y, Ebara S, Shiokawa D, et al. DNase gamma is the effector endonuclease for internucleosomal DNA fragmentation in necrosis. *PLoS One.* 2013;8(12):e80223.
3. Koyama R, Arai T, Kijima M, Sato S, Miura S, Yuasa M, et al. DNase gamma, DNase I and caspase-activated DNase cooperate to degrade dead cells. *Genes Cells.* 2016;21(11):1150-63.

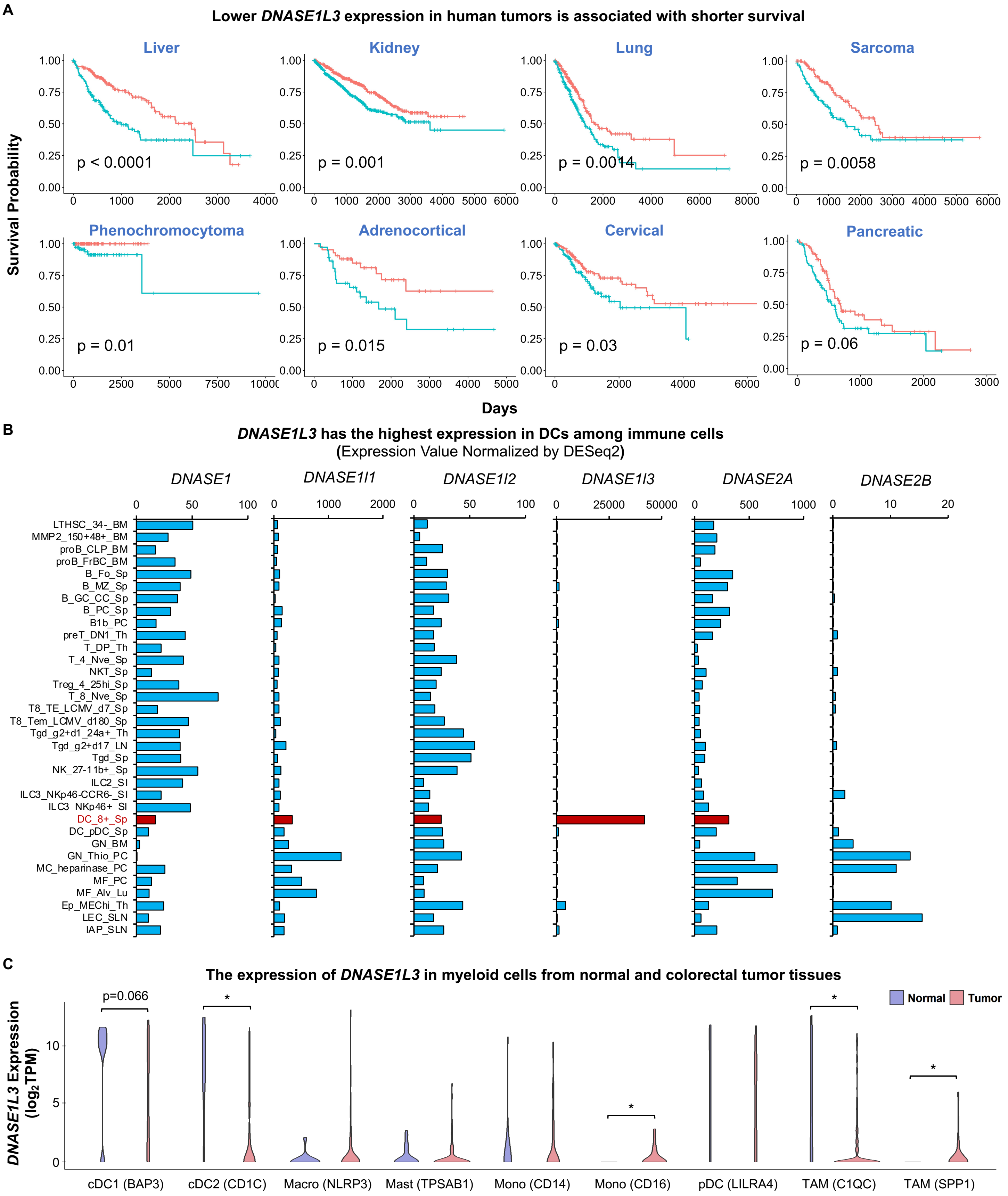

Figure S1. Downregulation of *DNASE1L3* in human tumors is associated with poor patient survival for many human cancer types.

(A) Kaplan-Meier survival probability curves for the indicated types of cancer patients from TCGA. Survival probability of the patients with DNASE1L3 expression level in tumors above the mean are shown in red (favorable) and of those with *DNASE1L3* expression level in tumors at or below the mean are shown in blue (unfavorable). P-value was computed based on the log-rank test of the survival distributions of high and low expression groups. (B) *DNASE1L3* has the highest expression in splenic CD8^{+} DCs among indicated immune cells. Normalized expression of indicated *DNASES* in immune cells was downloaded from http://rstats.immgen.org/. (C) *DNASE1L3* has the highest expression in cDCs among myeloid cells from normal human colonic tissues and its expression is downregulated in cDCs from colorectal tumors. Single-cell RNA-seq datasets of human colorectal tumors (18) were analyzed (Mann-Whitney test, *p<0.05).

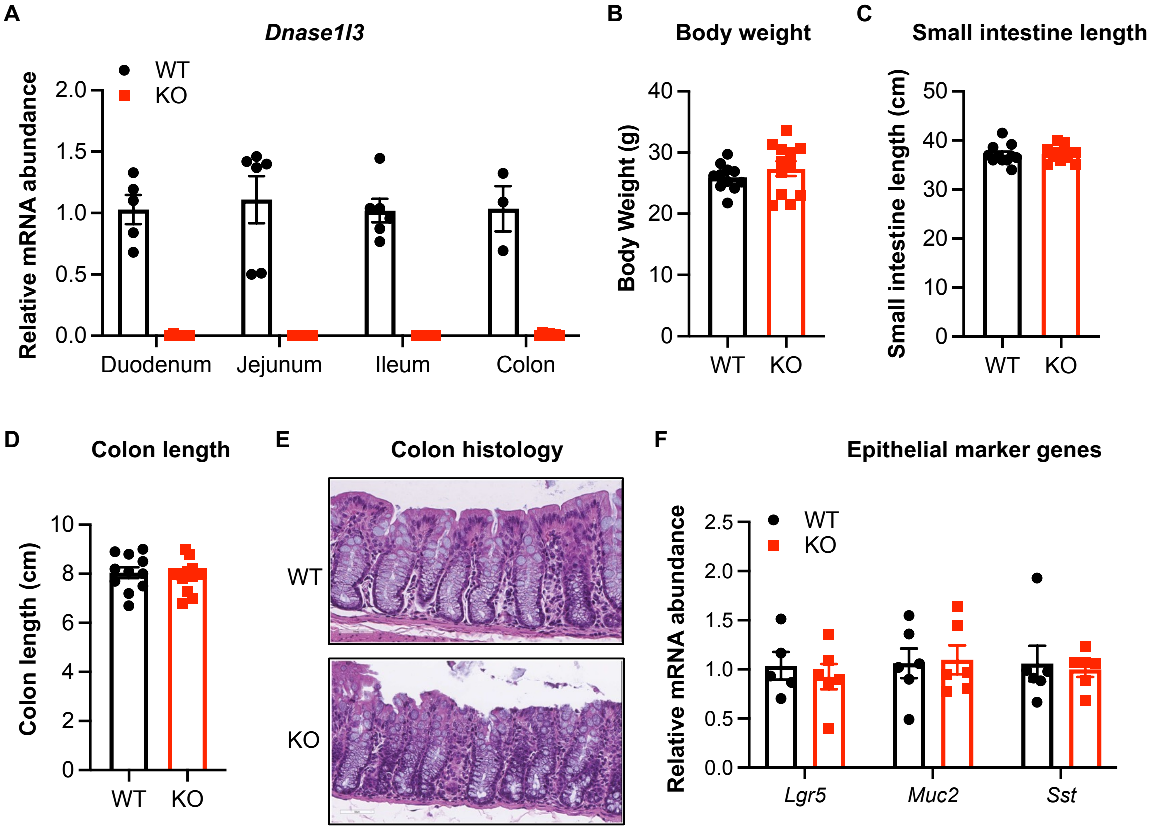

Figure S2: GI tract of *Dnase1l3* KO mice is phenotypically normal in basal conditions.

(A) *Dnase1l3* is deleted in all segments of the intestinal tract in *Dnase1l3* KO mice. Two- to three-month old Dnase1l3 WT and KO mice were fed with a regular chow diet. The abundance of Dnase1l3 mRNA in different segments of the intestinal tissue from *Dnase1l3* WT and KO mice was analyzed by qPCR (n=5, 6, 6, 3 WT and 6, 6, 6, 6 KO in each segment, respectively). (B-D) *Dnase1l3* KO mice have normal body weight (B), normal intestine length (C) and normal colon length (D) (n=11 WT and 12 KO). (E) *Dnase1l3* KO mice have normal colonic morphology. Representative H&E colonic sections from Dnase1l3 WT and KO mice are shown. (F) *Dnase1l3* KO mice have normal expression of stem (Lgr5), goblet (Muc), and endocrine (Sst) cell markers. The mRNA levels of indicated genes were analyzed by qPCR in colon of Dnase1l3 WT and KO mice (n=6 WT and 6 KO).

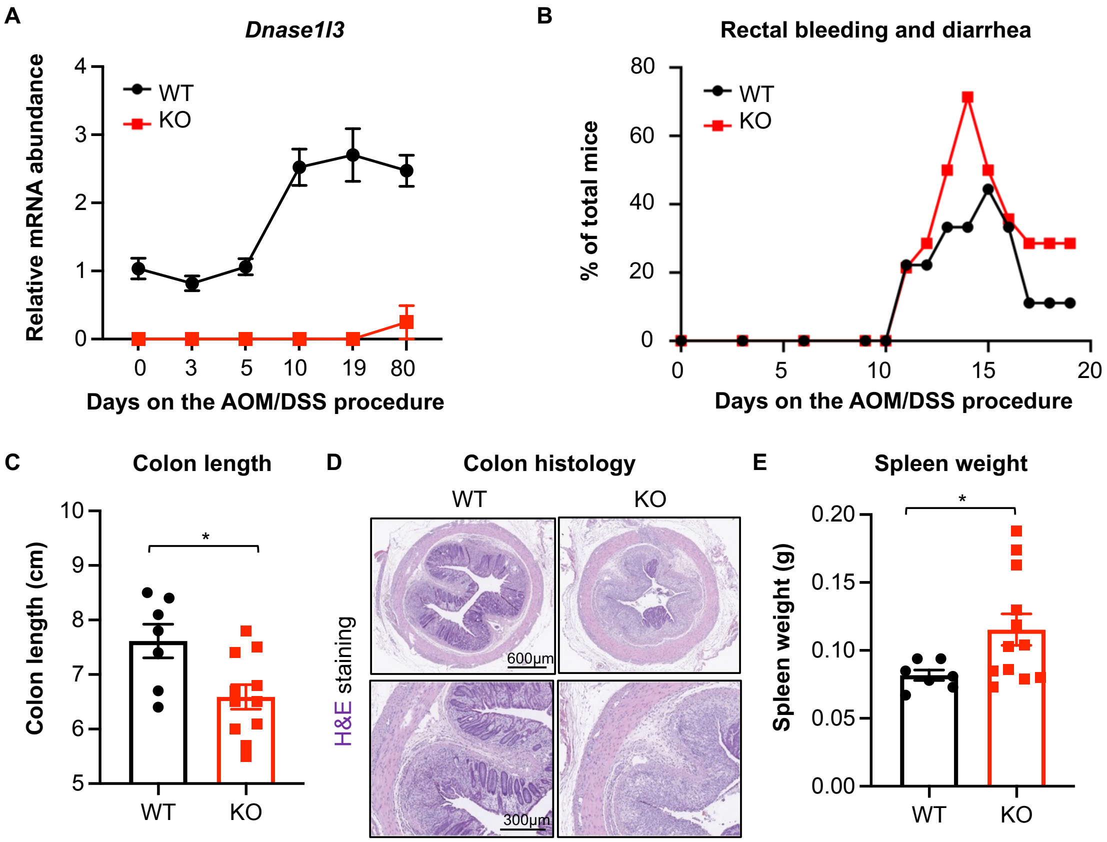

Figure S3. *Dnase1l3* deficiency impairs colonic tissue recovery after DSS treatment in AOM/DSS model.

Two- to three-month-old *Dnase1l3* WT and KO mice were subjected to AOM/DSS procedure as described in Methods and Figure 3A. (A) The expression of *Dnase1l3* during the AOM/DSS procedure (n=4, 5, 6, 10, 6, 12 WT and 4, 7, 7, 8, 12, and 10 KO in each time point, respectively). (B) Higher percentage of *Dnase1l3* KO mice display severe bleeding and diarrhea compared to WT mice during the first cycle of DSS treatment (n=7 WT and 14 KO). (C) *Dnase1l3* KO mice have shorter colon length compared to the WT mice at D19 (n=7 WT and 12 KO; Mann-Whitney test, *p<0.05). (D) *Dnase1l3* KO mice have impaired tissue morphology at the recovery stage (D19). Representative H&E staining images from the colon of WT and *Dnase1l3* KO mice are shown. (E) *Dnase1l3* KO mice have increased spleen weight compared to WT mice at the recovery stage (D19) (n=7 WT and 12 KO; Mann-Whitney test, *p<0.05).

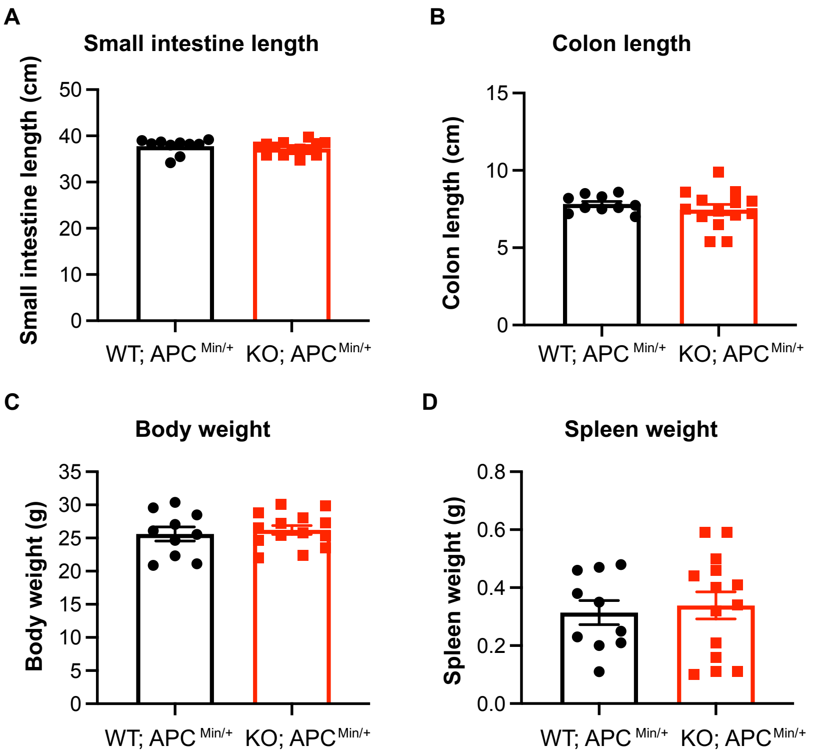

Figure S4. *Dnase1l3* deficiency in APC $^{Min/+}$ mice does not affect gross tissue morphology.

Dnase1l3$^{-/-}$;APC$^{min/+}$ mice have similar intestine length (A), colon length (B), body weight (C), and spleen weight (D) compared to *Dnase1l3$^{+/+}$;APC$^{min/+}$* mice (n=10 *Dnase1l3$^{+/+}$;APC$^{min/+}$* mice and 14 *Dnase1l3$^{-/-}$;APC$^{min/+}$* mice).

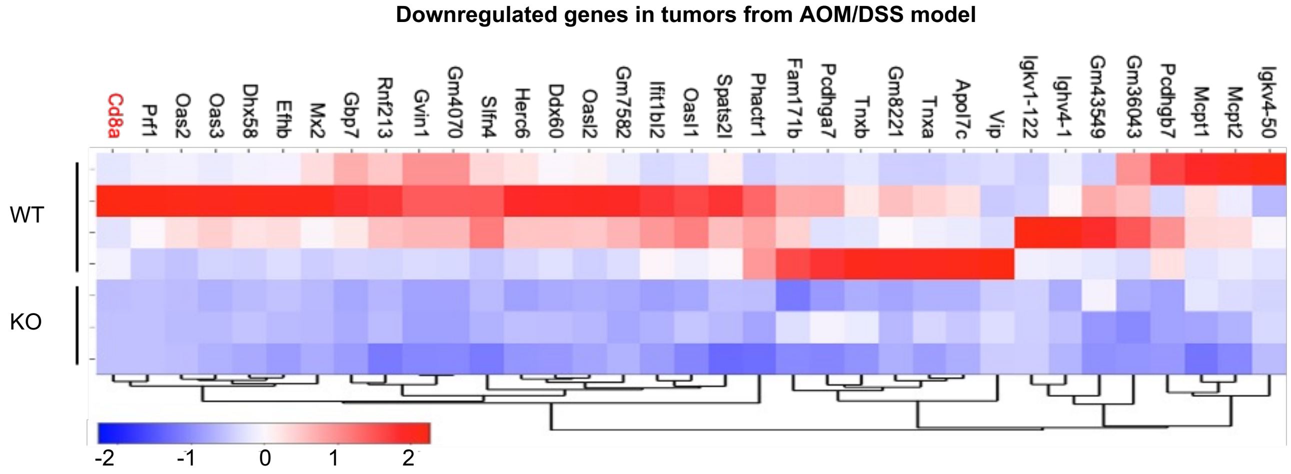

Figure S5. Colorectal tumors from *Dnase1l3* KO mice have reduced expression of multiple interferon pathway genes. Heatmap of the 35 down-regulated genes in the tumors isolated from WT and Dnase1l3 KO mice in the AOM/DSS colorectal cancer model.

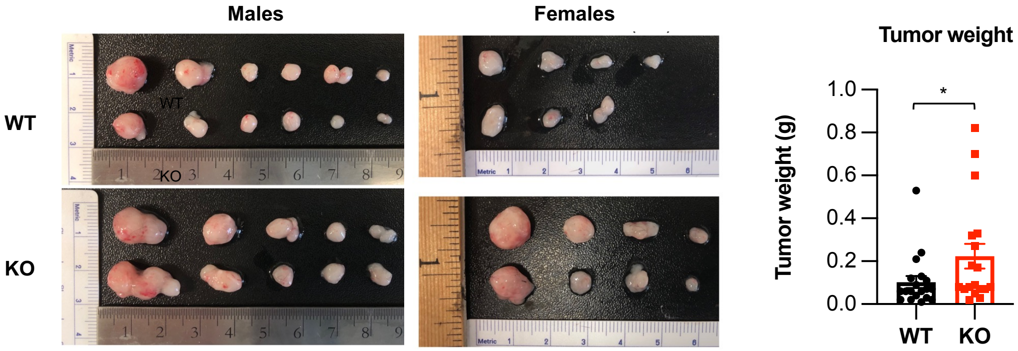

Figure S6. *Dnase1l3* deficiency increases the growth of MC38 tumor cells. 1 x 10^5 MC38 colon cancer cells were subcutaneously injected into 8-12 week-old WT or *Dnase1l3* KO mice and tumors were collected at Day 21. Weights of final MC38 tumors (n=19 tumors from WT and 18 tumors from KO from two additional independent experiments; Mann-Whitney test, *p<0.05).

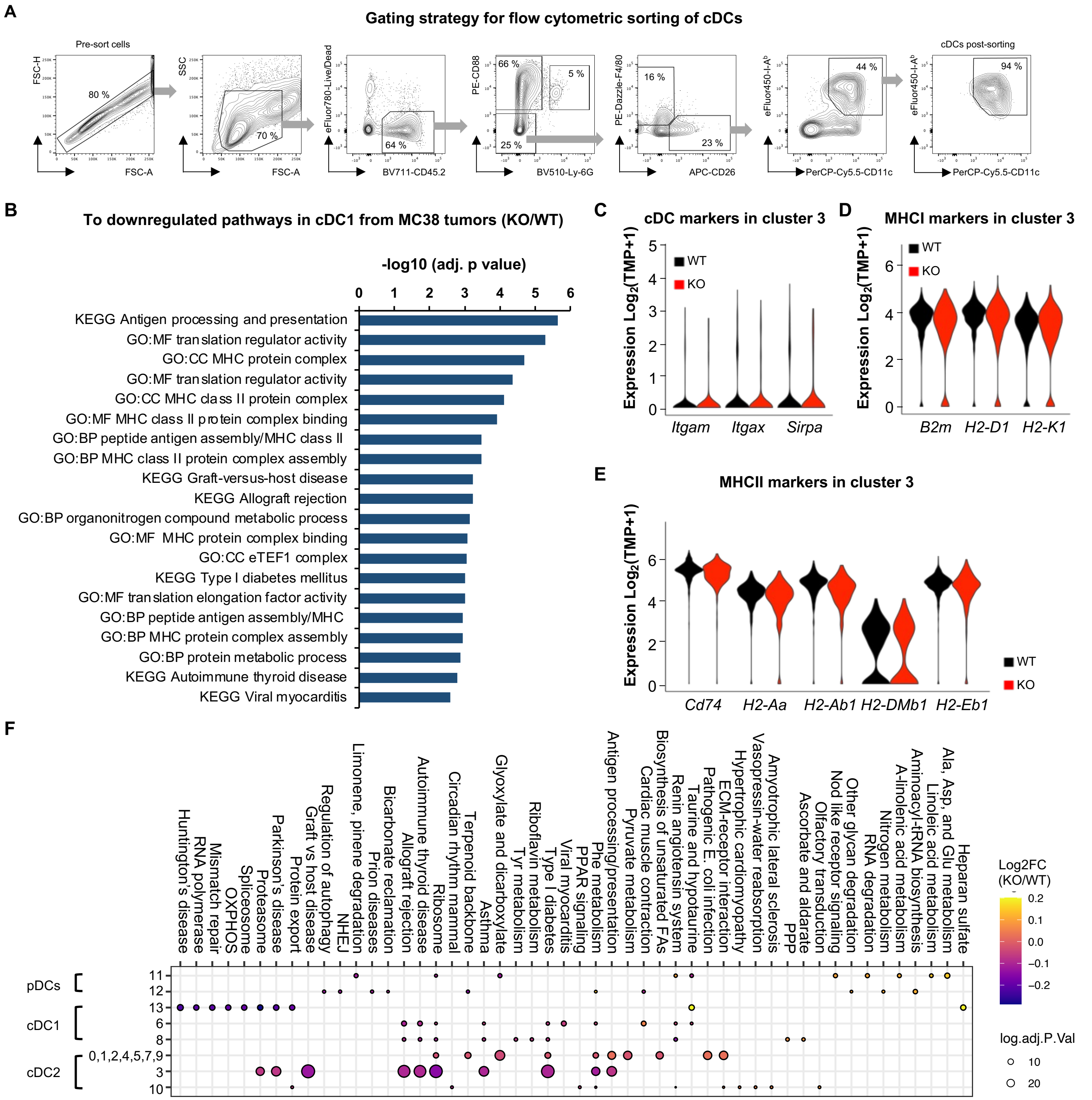

Figure S7. scRNA-seq analysis of the cDC populations in MC38 syngeneic colon tumors from *Dnase1l3* WT and KO mice.

(A) Gating strategy for flow cytometric sorting of cDCs isolated from subcutaneously grafted M38 tumors dissected 10 days after injection, and their purity post-sorting. Sorted cells were applied for scRNA-Seq analyses presented on Figure 6 and Figure S6B-S6E. (B) cDC1 display transcriptional downregulation in gene sets associated with antigen processing and presentation, and protein translation and expression. All DEGs (p adj <0.05, Log2FC<-0.2) from cDC1 from three clusters (6, 8, and 13) of cDC1 were analyzed for GO and pathway enrichment as described in Methods. (C) cDCs in cluster 3 have low expression levels of cDC markers. (D-E) cDCs in cluster 3 have high levels of MHCI (D) and MHCII genes (E). (F) GSVA analysis of transcriptomic alterations in cDCs from MC38 tumors in KO vs WT mice. Analysis was performed as described in Methods. Top 10 enriched KEGG pathways (7 upregulated and 3 downregulated) from each cell clusters are shown.

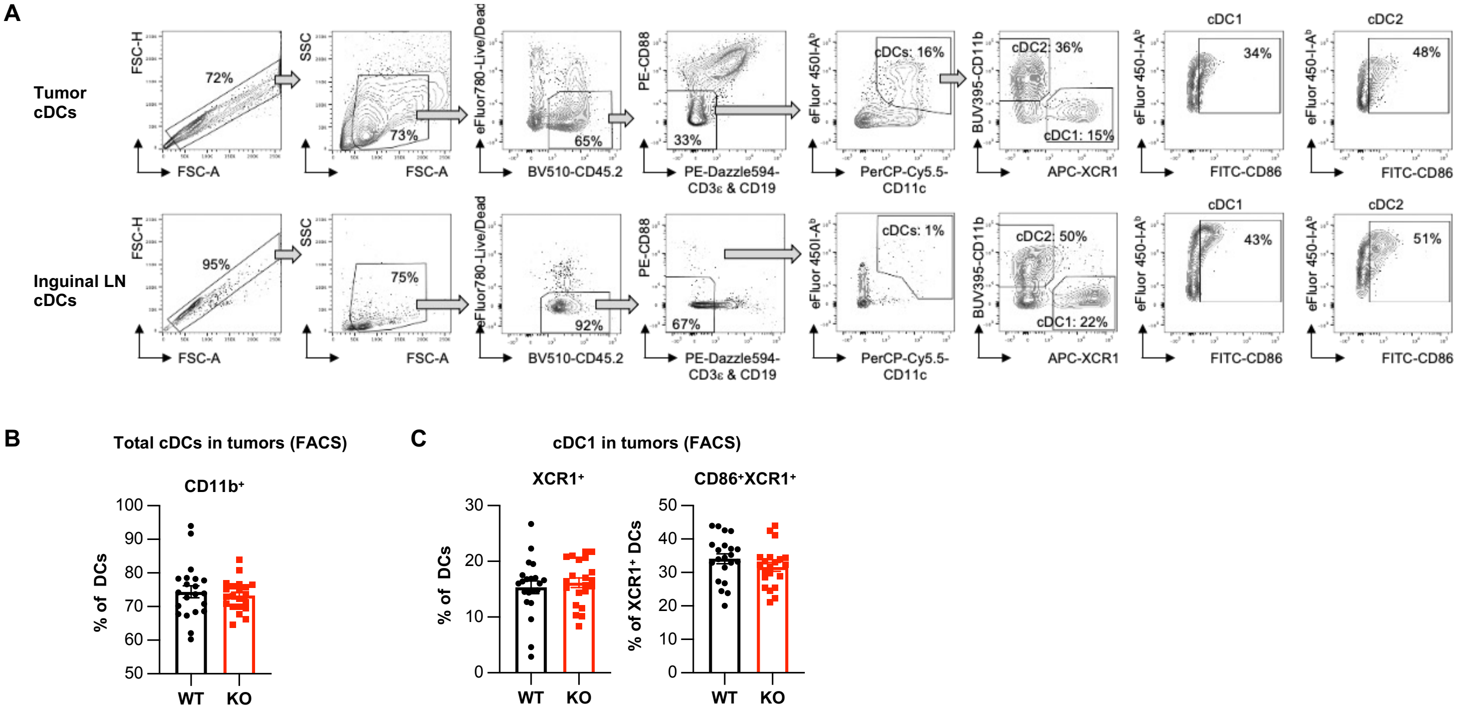

Figure S8. FACS analysis of cDCs in tumors and dLNs.

(A) Gating strategy for flow cytometric analysis of cDCs isolated from subcutaneously grafted M38 tumors or tumor-draining inguinal LNs. Data were presented on Figure 5I-5J, and B and C. (B) MC38 tumors from Dnase1l3 KO mice have normal abundance of infiltrating total cDCs (CD11b$^+$). (C) MC38 tumors from *Dnase1l3* KO mice have normal abundance of infiltrating total cDC1. For (B) and (C), Indicated immune cell populations in dLNs were analyzed by flow cytometry (n=16 WT and 16 KO from two independent experiments).

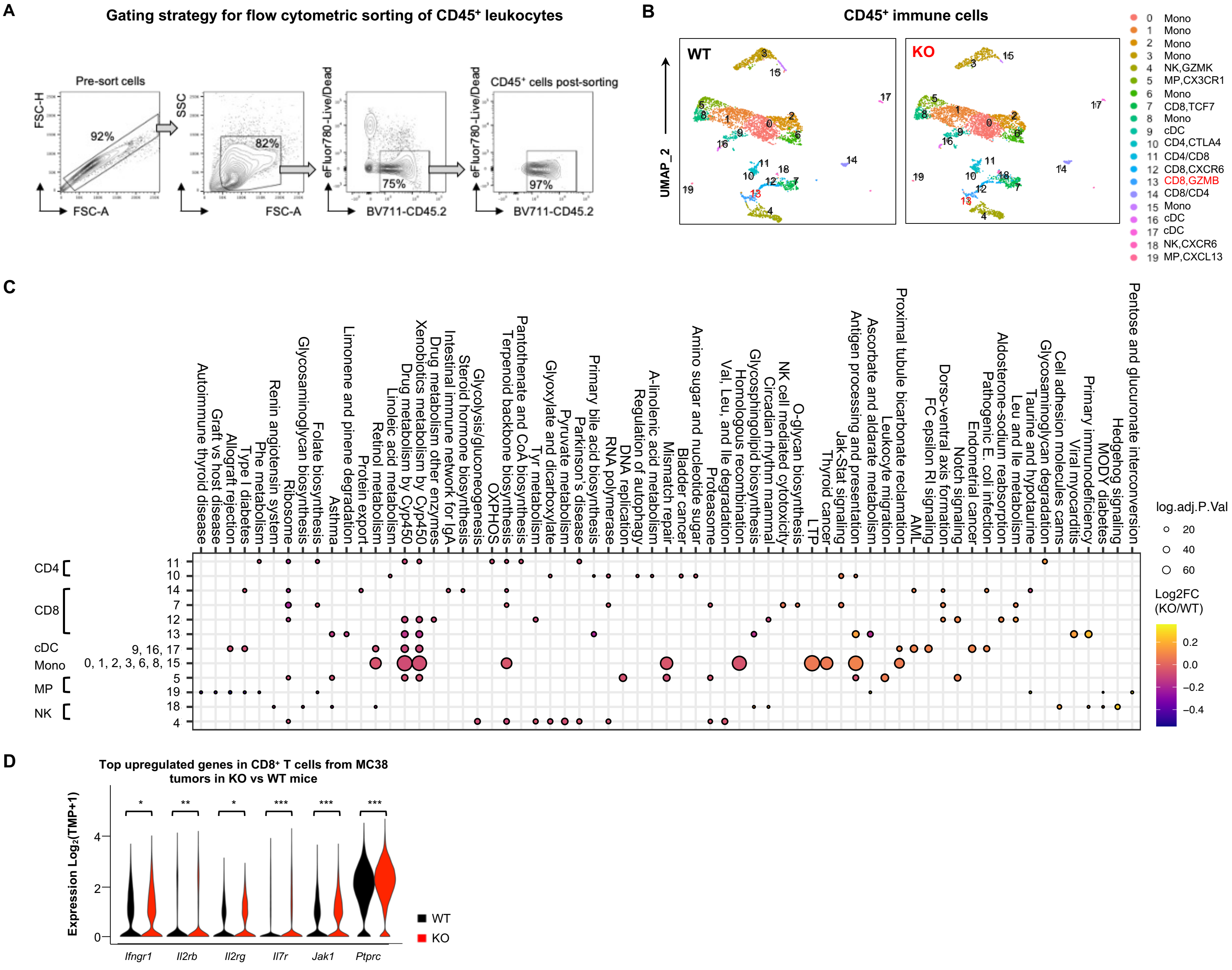

Figure S9. scRNA-seq analysis of the CD45+ populations in MC38 tumors from *Dnase1l3* WT and KO mice.

(A) Gating strategy for flow cytometric sorting of CD45+ leukocytes isolated from subcutaneously grafted M38 tumors, and their purity post-sorting. Sorted cells were applied for scRNA-Seq analyses presented on the rest of this figure. (B) UMAP analysis of 4,404 and 4,549 CD45⁺ single cells sampled from MC38 tumors from *Dnase1l3* WT and KO mice, respectively. (C) GSVA analysis of transcriptomic alterations in CD45⁺ immune cells from MC38 tumors in KO vs WT mice. Analysis was performed as described in Methods. Top 10 enriched KEGG pathways (7 upregulated and 3 downregulated) from each cell clusters are summarized. (D) CD8⁺ T cells from MC38 tumors in *Dnase1l3* KO mice have increased expression of several T cell activation and differentiation markers. Total CD45⁺ immune cells from MC38 tumors in *Dnase1l3* WT or KO mice were analyzed by scRNA-seq as described in Methods (Mann-Whitney test, *padj<0.05, **padj<0.01, ***padj<0.001).

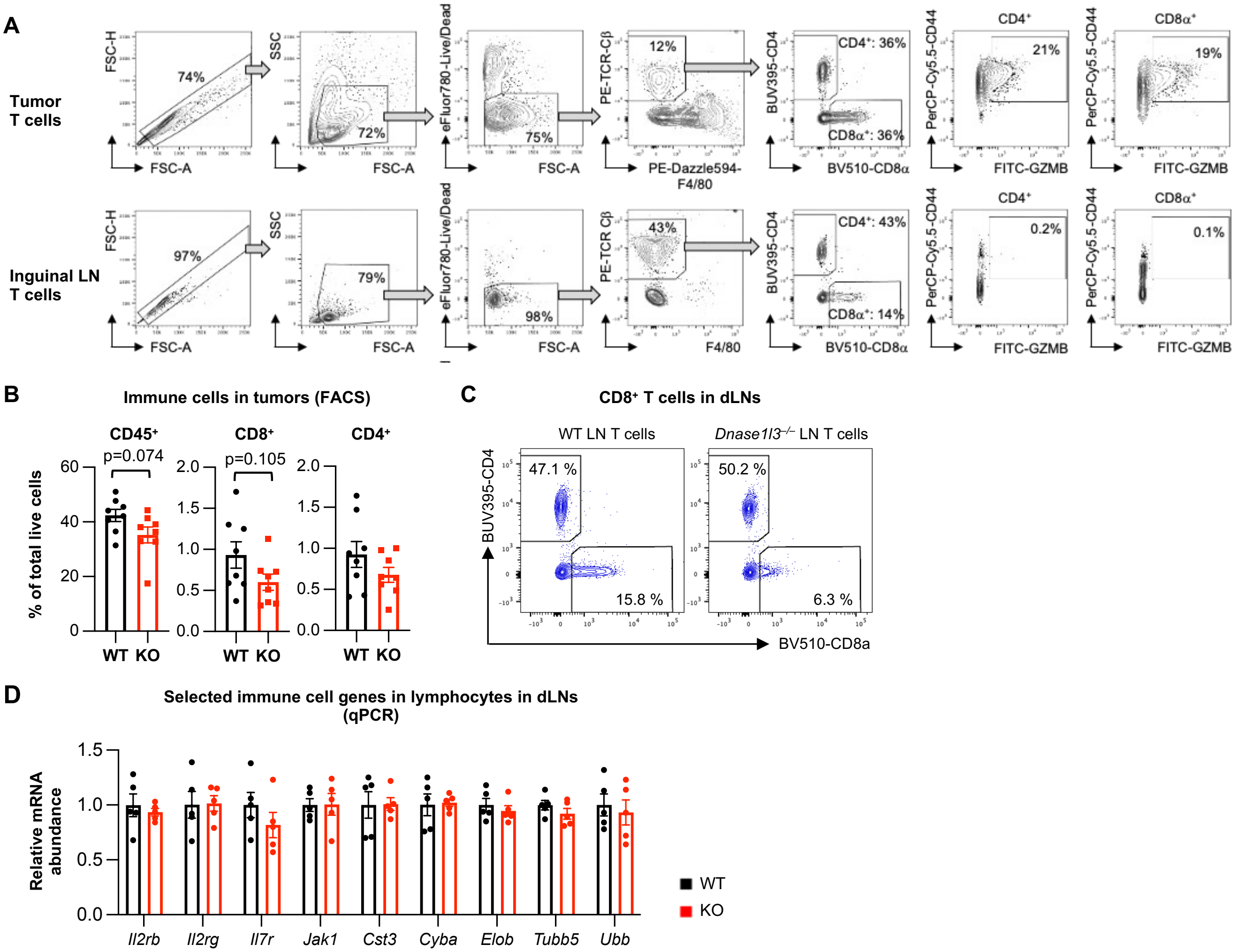

Figure S10. Tumors from *Dnase1l3* KO mice have reduced accumulation of immune cells.

(A) Gating strategy for flow cytometric analysis of T cells isolated from subcutaneously grafted M38 tumors or tumor-draining inguinal LNs. Data were presented on Figure 6C and 6D. (B) The abundance of indicated immune cells in MC38 tumors at an early stage of tumor development (14 days after inoculation). Indicated immune cell populations in isolated tumors were analyzed by flow cytometry (n=8 WT and 8 KO mice; Mann-Whitney test). (C) Representative FACS plots of $CD8^{+}$ T cells from MC38 tumors isolated from WT and KO mice (shown on Figure 6D, left). (D) The expression of indicated immune cell genes in isolated $CD45^{+}$ lymphocytes from dLNs was analyzed by qPCR (n=5 WT and 5 KO mice).
